# Plasticity in the Human Gut Microbiome Defies Evolutionary Constraints

**DOI:** 10.1128/mSphere.00271-19

**Published:** 2019-07-31

**Authors:** Andres Gomez, Ashok Kumar Sharma, Elizabeth K. Mallott, Klara J. Petrzelkova, Carolyn A. Jost Robinson, Carl J. Yeoman, Franck Carbonero, Barbora Pafco, Jessica M. Rothman, Alexander Ulanov, Klara Vlckova, Katherine R. Amato, Stephanie L. Schnorr, Nathaniel J. Dominy, David Modry, Angelique Todd, Manolito Torralba, Karen E. Nelson, Michael B. Burns, Ran Blekhman, Melissa Remis, Rebecca M. Stumpf, Brenda A. Wilson, H. Rex Gaskins, Paul A. Garber, Bryan A. White, Steven R. Leigh

**Affiliations:** a Department of Animal Science, University of Minnesota, Twin Cities, St. Paul, Minnesota, USA; b Department of Anthropology, Northwestern University, Evanston, Illinois, USA; c Institute of Vertebrate Biology, The Czech Academy of Sciences, Brno, Czech Republic; d Institute of Parasitology, Biology Centre of the Czech Academy of Sciences, Ceske Budejovice, Czech Republic; e Liberec Zoo, Liberec, Czech Republic; f Department of Anthropology, University of North Carolina, Wilmington, North Carolina, USA; g Department of Animal and Range Sciences, Montana State University, Bozeman, Montana, USA; h Department of Nutrition & Exercise Physiology, Elson S. Floyd College of Medicine, Washington State University, Spokane, Washington, USA; i Department of Pathology and Parasitology, Faculty of Veterinary Medicine, University of Veterinary and Pharmaceutical Sciences Brno, Brno, Czech Republic; j Department of Anthropology, Hunter College of CUNY and New York Consortium in Evolutionary Primatology (NYCEP), New York, New York, USA; k Metabolomics Center, Roy J. Carver Biotechnology Center, University of Illinois at Urbana-Champaign, Champaign, Illinois, USA; l Department of Anthropology, University of Nevada, Las Vegas, Nevada, USA; m Department of Anthropology, Dartmouth College, Hanover, New Hampshire, USA; n Central European Institute for Technology (CEITEC), University of Veterinary and Pharmaceutical Sciences Brno, Brno, Czech Republic; o World Wildlife Fund, Dzanga-Sangha Protected Areas, Bayanga, Central African Republic; p J. Craig Venter Institute, La Jolla, California, USA; q Department of Biology, Loyola University Chicago, Chicago, Illinois, USA; r Department of Genetics, Cell Biology, and Development, University of Minnesota, Twin Cities, Minneapolis, Minnesota, USA; s Department of Anthropology, Purdue University, West Lafayette, Indiana, USA; t Carl Woese Institute for Genomic Biology, University of Illinois at Urbana-Champaign, Champaign, Illinois, USA; u Department of Anthropology, University of Illinois at Urbana-Champaign, Champaign, Illinois, USA; v Department of Microbiology, University of Illinois at Urbana-Champaign, Champaign, Illinois, USA; w Department of Animal Sciences, University of Illinois at Urbana-Champaign, Champaign, Illinois, USA; x Department of Anthropology, University of Colorado, Boulder, Colorado, USA; y Konrad Lorenz Institute for Evolution and Cognition Research, Klosterneuburg, Austria; University of Wisconsin-Madison

**Keywords:** evolution, microbiome, primate

## Abstract

The gut microbiome of primates, including humans, is reported to closely follow host evolutionary history, with gut microbiome composition being specific to the genetic background of its primate host. However, the comparative models used to date have mainly included a limited set of closely related primates. To further understand the forces that shape the primate gut microbiome, with reference to human populations, we expanded the comparative analysis of variation among gut microbiome compositions and their primate hosts, including 9 different primate species and 4 human groups characterized by a diverse set of subsistence patterns (*n* = 448 samples). The results show that the taxonomic composition of the human gut microbiome, at the genus level, exhibits increased compositional plasticity. Specifically, we show unexpected similarities between African Old World monkeys that rely on eclectic foraging and human populations engaging in nonindustrial subsistence patterns; these similarities transcend host phylogenetic constraints. Thus, instead of following evolutionary trends that would make their microbiomes more similar to that of conspecifics or more phylogenetically similar apes, gut microbiome composition in humans from nonindustrial populations resembles that of generalist cercopithecine monkeys. We also document that wild cercopithecine monkeys with eclectic diets and humans following nonindustrial subsistence patterns harbor high gut microbiome diversity that is not only higher than that seen in humans engaging in industrialized lifestyles but also higher compared to wild primates that typically consume fiber-rich diets.

**IMPORTANCE** The results of this study indicate a discordance between gut microbiome composition and evolutionary history in primates, calling into question previous notions about host genetic control of the primate gut microbiome. Microbiome similarities between humans consuming nonindustrialized diets and monkeys characterized by subsisting on eclectic, omnivorous diets also raise questions about the ecological and nutritional drivers shaping the human gut microbiome. Moreover, a more detailed understanding of the factors associated with gut microbiome plasticity in primates offers a framework to understand why humans following industrialized lifestyles have deviated from states thought to reflect human evolutionary history. The results also provide perspectives for developing therapeutic dietary manipulations that can reset configurations of the gut microbiome to potentially improve human health.

## Abstract (precis)

The results of this study indicate a discordance between gut microbiome composition and evolutionary history in primates, calling into question previous notions about host genetic control of the primate gut microbiome. Microbiome similarities between humans consuming nonindustrialized diets and monkeys characterized by subsisting on eclectic, omnivorous diets also raise questions about the ecological and nutritional drivers shaping the human gut microbiome. Moreover, a more detailed understanding of the factors associated with gut microbiome plasticity in primates offers a framework to understand why humans following industrialized lifestyles have deviated from states thought to reflect human evolutionary history. The results also provide perspectives for developing therapeutic dietary manipulations that can reset configurations of the gut microbiome to potentially improve human health.

## INTRODUCTION

Identifying the factors that drive the composition and function of the human gut microbiome has been the subject of extensive research in the microbiome field. Comparative models have explored the gut microbiome of human populations across diverse subsistence gradients (1–4). These models have also established evolutionary parallels between humans and nonhuman primates (5, 6), providing valuable insights for understanding the intersections between host genetics, diet, and lifestyle in shaping the human microbiome.

Although it remains generally assumed that phylogenetic conservatism likely associated with host physiology outweighs diet in determining primate gut microbiomes (6–8), it has also been shown that subsistence strategies and diet are major drivers of the gut microbiome of humans (9–12) and nonhuman primates (13–15). However, the existing comparative models have considered only humans under different subsistence strategies (4), closely related primate species consuming similar diets in the wild (16), or primates consuming controlled diets in captivity (17).

In an effort to better define the factors shaping the current taxonomic composition of the human gut microbiome, we used an expanded comparative model across a phylogenetically and ecologically diverse set of primate species, including great apes, Old World monkeys, New World monkeys, and human populations with markedly different subsistence patterns. We hypothesize that the human gut microbiome is plastic, and that it deviates from the phylogenetic conservatism proposed before (6). In reflecting this plasticity, we propose that the gut microbiome of humans following traditional subsistence practices (hunter-gathering and traditional agriculture) share taxonomic and diversity signatures with distantly related nonhuman primates.

## RESULTS

A total of 392 fecal samples of anthropoid primates were collected in different locations across Africa, Central America, and Mexico, including African great apes (mountain gorillas [*n* = 48], western lowland gorillas [*n* = 191], and Central African chimpanzees [*n* = 10]), Old World monkeys (olive baboons [*n* = 4], geladas [*n* = 7], agile mangabeys [*n* = 10], and vervets [*n* = 23]), as well as New World monkeys (black howlers [*n* = 33] and captive tufted capuchins [*n* = 4]). Our human samples are derived from multiple groups, including hunter-gatherers (The BaAka, *n* = 28) and traditional agriculturalists (The Bantu, *n* = 29) from the Dzanga Sangha Protected Areas, Central African Republic, and western researchers working at the same field site for 3 to 6 months (*n* = 5) (see Table S1 in the supplemental material for details). DNA was extracted from each sample, and the V1-V3 hypervariable region of the 16S rRNA bacterial gene was sequenced to determine bacterial community composition across all primate groups. 16S rRNA sequences generated from fecal samples of U.S. participants (*n* = 56) of the Human Microbiome Project (termed U.S.-HMP) (18, 19) were added to the comparative analyses. Sequence data were processed and collapsed according to bacterial genus presence and abundance across all samples.

10.1128/mSphere.00271-19.7 TABLE S1 Details of samples included in this study. Download Table S1, DOCX file, 0.06 MB. Copyright © 2019 Gomez et al. 2019 Gomez et al. This content is distributed under the terms of the Creative Commons Attribution 4.0 International license.

### Phylogenetically distant primates share similar gut microbiomes.

**FIG 1:**
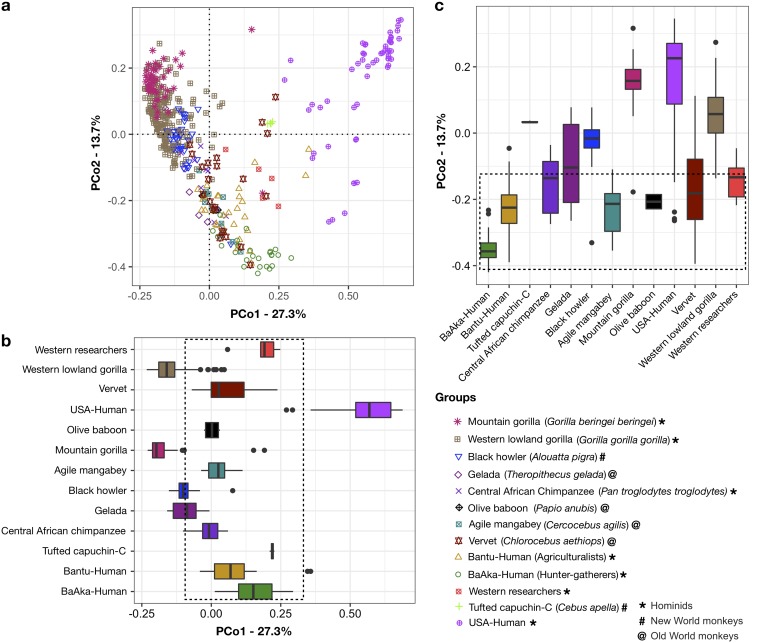
Gut bacterial community composition differs across multiple primate species. (a) Bray-Curtis-based principal coordinate analysis showing different bacterial community composition in fecal samples of all primate species analyzed (*R*^2^ = 0.49 and *P* < 0.001 by PERMANOVAs). Each symbol represents the fecal microbiome composition, at the genus level, of a different primate species. (b and c) Boxplots showing ordination scores of each sample along PCo1 (b) and PCo2 (c). The dotted boxes are drawn to enclose groups where the medians are not statistically different in ordination scores (*P* > 0.05) according to Kruskal-Wallis multiple comparisons. Western researchers, subjects working in the Dzanga Sangha Protected Areas, Central African Republic; capuchin-C, captive tufted capuchins; USA-Human, U.S. subjects, part of the Human Microbiome Project. The bottom right corner shows colored symbols for every species in the PCoA ordination in panel a, in ascending order according to median scores along PCo1.

A Bray-Curtis distance ordination analysis (principal coordinate analysis [PCoA] of genus-level relative abundance tables) revealed significant stratification of the fecal microbiome of each primate species (*R*^2^ = 0.49 and *P* < 0.001 according to permutational multivariate analyses of variance [PERMANOVAs]) (Fig. 1a). Species-specific arrangement of primate gut microbiomes has been observed previously (6) and was replicated here based on diverse distance metrics, including UniFrac (Fig. S1a to c). Nonetheless, our data also revealed significant stratification of the human gut microbiome depending on either geographical origin or subsistence strategy. For example, the gut microbiomes of U.S.-HMP subjects deviated significantly from those of all other primates, including all other human groups. In contrast, the gut microbiome composition of hunter-gatherers and traditional agriculturalists clustered closely in distance with those of all other nonhuman primates, specifically with those of vervets, mangabeys, and baboons. This distance overlap can be seen in Fig. 1b and c, where the dotted box indicates no significant difference in median distance ordination scores along principal coordinates one and two (PCo1 and PCo2), between the groups following traditional subsistence practices, and the vervet, mangabey, and baboon monkeys (*P* > 0.05 by Wilcoxon rank sum test).

10.1128/mSphere.00271-19.1 FIG S1 Gut bacterial community composition differs across multiple primate species. (a to c) Canberra, UniFrac (weighted), and UniFrac (unweighted) distance-based principal coordinate analysis showing different bacterial community composition in fecal samples of all primate species analyzed. Each symbol represents the fecal microbiome composition of a different primate species. USA, U.S. subjects, part of the Human Microbiome Project; western researchers, subjects working in an African field site. Download FIG S1, TIFF file, 2.0 MB. Copyright © 2019 Gomez et al. 2019 Gomez et al. This content is distributed under the terms of the Creative Commons Attribution 4.0 International license.

These nonhuman primates are African monkeys of the subfamily *Cercopithecinae*, whose molecular divergence from the superfamily *Hominoidea* (apes and humans) is estimated at 23 million years ago (20). The results showed that hunter-gatherers and traditional agriculturalists shared more microbiome compositional features with African cercopithecines than they did with U.S. humans and even with more phylogenetically related primates, such as gorillas or chimpanzees, whose divergence from humans occurred from 6 to 9 million years ago. Moreover, the gut microbiome of western researchers (from the Czech Republic, the United Kingdom, and the United States) working in the Dzanga Sangha Protected areas of the Central African Republic, who transitioned to the lifestyles and diets of the traditional agriculturalists during their stay in the field from 3 to 6 months, showed microbiome trends similar to those seen in these traditional populations. The close similarities in gut microbiome composition between humans following traditional subsistence practices and the cercopithecine monkeys are seen not only in multivariate space, along two principal coordinates (Fig. 1 and Fig. S1), but also in terms of unweighted UniFrac distances (Fig. S2a to d).

10.1128/mSphere.00271-19.2 FIG S2 Similarities in bacterial community composition between different human groups and nonhuman primates. The boxplots show how similar (or dissimilar) the fecal microbiome of the BaAka hunter gatherers (a), Bantu agriculturalists (b), western researchers working in an African field site (c), and (d) U.S. humans are from those of different nonhuman primates. The dotted boxes are drawn to enclose groups where the medians are not statistically different (*P* > 0.05) according to Kruskal-Wallis multiple comparisons. BaAka, BaAka humans; Bantu, Bantu humans; baboon, olive baboon; capuchin-C, tufted capuchin-C; chimp, Central African chimpanzee; gorilla, western lowland gorilla; howler, black howler; mangabey, agile mangabey; Mountain, mountain gorilla; USA, U.S. humans; WR, western researchers. Download FIG S2, TIF file, 0.7 MB. Copyright © 2019 Gomez et al. 2019 Gomez et al. This content is distributed under the terms of the Creative Commons Attribution 4.0 International license.

**FIG 2:**
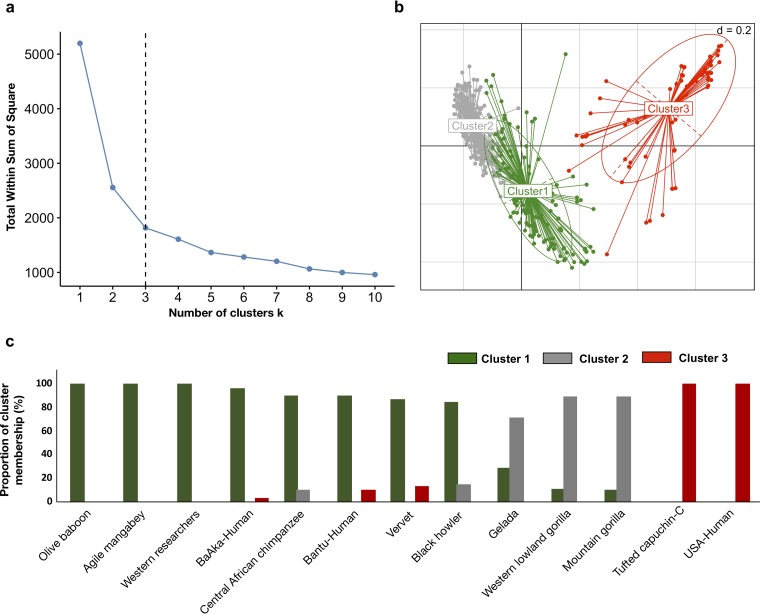
Gut bacterial communities of different primate species are classified into three different clusters. (a) Partition around medoids (PAM) clustering analysis shows that total within sums of squares (classification error) diminishes significantly when considering three clusters, with no significant further error reductions. (b) Bray-Curtis PCoA ordination showing the presence of three clusters classifying gut bacterial community composition across different primate species. Ellipses show confidence intervals (95% standard errors) in multivariate space, and each dot represents the fecal sample of a given primate. (c) Bar plots showing the proportion of individuals within each primate species classified into each of the three clusters detected.

To validate these grouping patterns, an unsupervised cluster analysis was performed (partitioning around medoids, or PAM, clustering). Average silhouette width indicated efficient stratification of the microbiome composition of the primate groups studied, dividing them into two main clusters (Fig. S3a). These clusters strongly reflect dissimilarity between U.S. subjects and all other nonhuman primates and humans (Fig. S3b). However, upon closer inspection and using a different PAM feature (total within sums of squares or classification error), better classification accuracy was detected when the data were divided into three clusters (Fig. 2a) and corroborated grouping of samples based on the similarity dynamics identified previously (Fig. 1 and 2b). For example, all U.S.-HMP subjects constituted a separate cluster, along with all captive capuchins; this group was designated cluster three. The vast majority of mountain and lowland gorillas (>∼90%) clustered together with most geladas (>∼75%) and were designated cluster two. Most human hunter-gatherers and traditional agriculturalists (>∼90%) and all western researchers clustered with all baboons, mangabeys, vervets, and chimpanzees (>∼90%) and most howler monkeys (>∼80%) and were grouped into cluster one (Fig. 2c). Cluster membership was corroborated through a randomForest classification procedure (area under the curve of 0.986, out-of-bag estimate of error rate of 6.03%; cluster one, 11%; cluster two, 3%; cluster three,  3%).

10.1128/mSphere.00271-19.3 FIG S3 Gut bacterial communities of different primate species are classified into clusters. (a) Partition around medoids (PAM) clustering analysis shows that average silhouette width (a proxy for classification accuracy) is higher when considering two clusters, with no significant further error reductions after selecting 3 clusters. (b) Bray-Curtis PCoA ordination showing the presence of two clusters classifying gut bacterial community composition across different primate species. Ellipses show confidence intervals (95% standard errors) in multivariate space, and each dot represents the fecal sample of a given primate. Download FIG S3, TIF file, 0.8 MB. Copyright © 2019 Gomez et al. 2019 Gomez et al. This content is distributed under the terms of the Creative Commons Attribution 4.0 International license.

This cluster assortment of gut microbiome composition in the primates analyzed does not correspond with the pattern of primate phylogeny. Although this may be an obvious observation, to show this mismatch, we generated an unrooted 16S rRNA-Bray-Curtis distance tree based on average genus abundances in the gut microbiome of the primate groups analyzed and contrasted it with a tree generated with representative host mitochondrial DNA (mtDNA) sequences available in databases (Fig. S4). This analysis clearly shows that the topology of the two trees, 16S rRNA and mtDNA based, is discordant.

10.1128/mSphere.00271-19.4 FIG S4 Unrooted trees showing a mismatch between 16S rRNA-based microbiome profiles of each fecal sample analyzed and representative mtDNA sequences of the primate hosts. (a) Unrooted phylogenetic tree generated using ClustalW-based multiple-sequence alignment of mtDNA sequences of each primate species shows separate clades for hominids, New World monkeys, and Old World monkeys. (b) Bray-Curtis-based hierarchical clustering based on average genus abundances (fecal 16S rRNA data) for each primate group does not follow the same trend. Download FIG S4, TIFF file, 2.0 MB. Copyright © 2019 Gomez et al. 2019 Gomez et al. This content is distributed under the terms of the Creative Commons Attribution 4.0 International license.

### Taxonomic and diversity convergence in the gut microbiome of phylogenetically distant primates.

**FIG 3:**
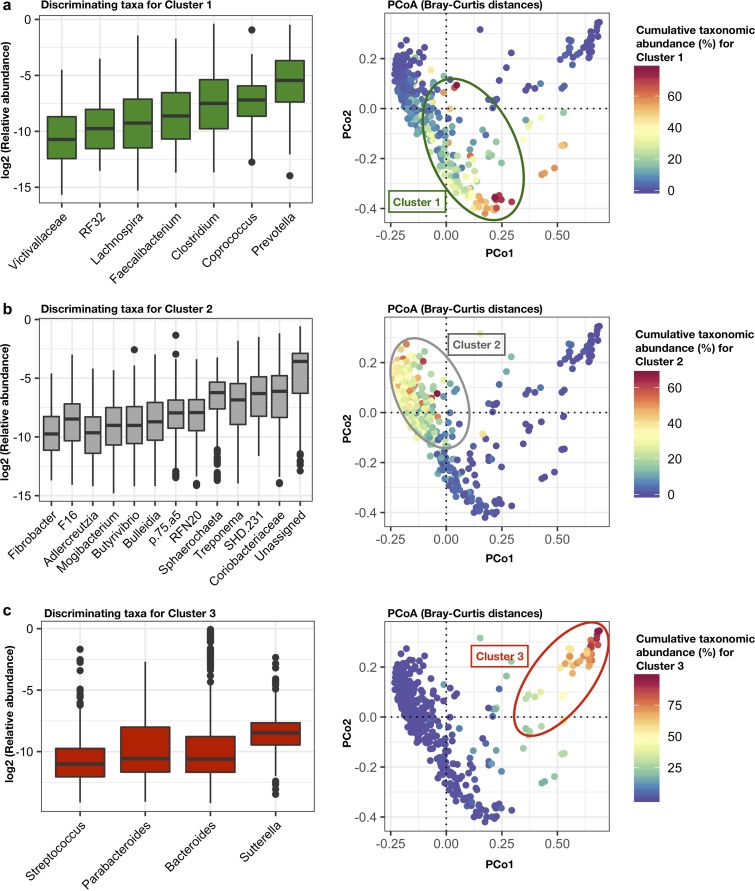
Specific gut bacterial taxonomic signatures characterize cluster membership across different primate species. (a to c, left) Boxplot showing the relative abundance (log transformed) of differentially abundant genera detected in each cluster (RandomForest mean decreased accuracy of >9; indicator value, >0.5; *P* < 0.05; Kruskal-Wallis tests, *q* < 0.05). (a to c, right) Bray-Curtis distance PCoA ordination colored by the cumulative relative abundance of taxonomic signatures shaping each cluster.

We used a combination of randomForest analyses and its mean decrease accuracy index (>9), along with species indicator analysis (indicator value of >0.5, *P* < 0.05), and Kruskal-Wallis test (multiple comparisons, *q* < 0.05) (Table S2) to identify the most representative genera of each cluster. The abundance of each cluster-specific bacterial genus was combined to show cumulative taxonomic signatures shaping each cluster. For instance, cluster one was primarily dominated by abundances of *Prevotella*, followed by *Coprococcus*, *Clostridium*, *Faecalibacterium*, *Lachnospira*, RF32, and unclassified *Victivallaceae* (Fig. 3a). Members of cluster two were mainly characterized by showing high abundance of unknown bacteria, followed by unknown *Coriobacteriaceae*, SDH 231, unknown *Sphaerochaeta*, *Treponema*, RFN20, p75a5, *Buleidia*, *Butyrivibrio*, *Mogibacterium*, *Aldercreutzia*, F16, and *Fibrobacter* (Fig. 3b). Finally, *Streptococcus*, *Parabacteroides*, *Bacteroides*, and *Sutterella* mainly distinguished members of cluster three (Fig. 3c).

10.1128/mSphere.00271-19.8 TABLE S2 List of significantly discriminating taxa belonging to each cluster. Download Table S2, DOCX file, 0.02 MB. Copyright © 2019 Gomez et al. 2019 Gomez et al. This content is distributed under the terms of the Creative Commons Attribution 4.0 International license.

**FIG 4:**
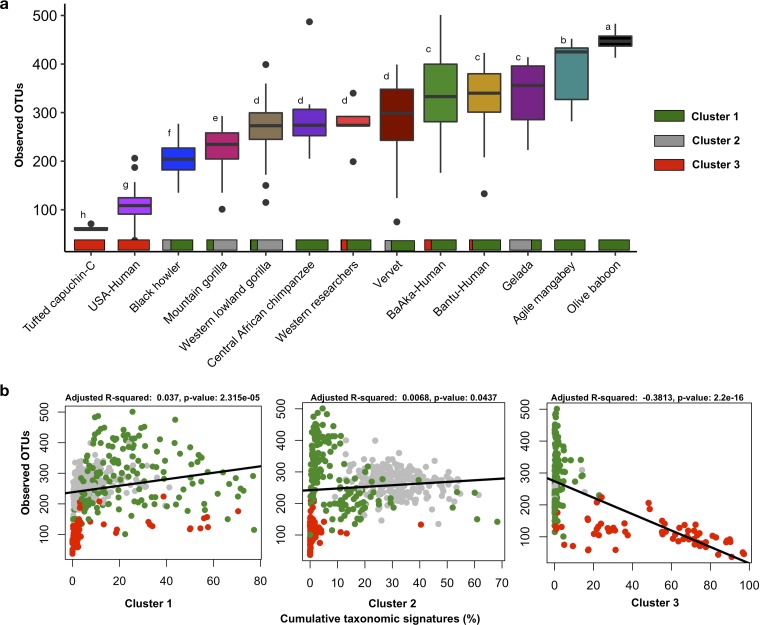
Number of observed taxonomic units in the gut microbiome of different primate species is dependent on cluster membership. (a) Boxplots showing number of observed OTUs in the fecal microbiome of different primate species according to cluster membership. Different letters denote significant differences according to FDR-adjusted Kruskal-Wallis tests. Number of observed OTUs was normalized (rarefied) at 1,000 reads per sample. (b) The number of observed OTUs in the fecal microbiome of different primate species is correlated with the abundance of cumulative taxonomic signatures characterizing each cluster.

We next explored alpha diversity in the context of cluster membership. Most members of cluster one, particularly baboons, mangabeys, traditional agriculturalists, and hunter-gatherers, showed the greatest number of observed taxonomic units in their fecal microbiomes. They were followed by members of cluster two, the highly folivorous/herbivorous mountain and lowland gorillas, which exhibited significantly lower numbers of observed taxa (*P* < 0.05) (Fig. 4a). Black howler monkeys, which emphasize a seasonally leaf-based diet balanced with some fruits (21), had numbers of observed taxa similar to those seen in the two gorilla species despite being mostly assigned to cluster one. Likewise, geladas, mostly assigned to cluster two, showed parity with the human groups following traditional lifestyles (agriculturalists and hunter-gatherers) (Fig. 4a). These alpha diversity results were replicated using other metrics, such as Chao1 and Shannon index, and both with rarefied and unrarefied data (Fig. S5a to f). U.S.-HMP subjects and captive capuchins, all members of cluster three, showed the lowest taxonomic richness in their gut microbiomes (Fig. 4a).

10.1128/mSphere.00271-19.5 FIG S5 Bacterial diversity in the gut microbiome of different primate species is dependent on cluster membership. (a to f) Boxplots showing that bacterial diversity in the fecal microbiome of different primate species varies according to cluster membership. Panels a to c show different alpha diversity indices (observed, Chao1, and Shannon) calculated on rarefied data (1,000 reads per sample), and panels d and e show the same indices in unrarefied data. The dotted boxes are drawn to enclose groups where the medians are not statistically different (*P* > 0.05) according to Kruskal-Wallis multiple comparisons. Lower side bars (*x* axis) indicate cluster membership. Download FIG S5, TIFF file, 7.9 MB. Copyright © 2019 Gomez et al. 2019 Gomez et al. This content is distributed under the terms of the Creative Commons Attribution 4.0 International license.

As a way to understand factors related to increased and decreased microbiome diversity among members of clusters one and two, respectively, we explored the hypothesis that the unique richness patterns observed within each primate cluster are correlated with the abundance of cluster-specific taxonomic features. As such, we analyzed the linear relationship between the number of observed taxa and the abundance of cumulative taxonomic signatures characterizing each cluster. For instance, the cumulative abundance of *Prevotella*, and other cluster one-specific genera, was concordant with an increase in microbiome richness (*R*^2^ = 0.03, *P* = 2.31e−05) (Fig. 4b, left). The cumulative abundance of genera characterizing cluster 3 (*Streptococcus*, *Parabacteroides*, *Bacteroides*, and *Sutterella*) was negatively correlated with the number of taxonomic units observed among members of this cluster (*R*^2^ = −0.38, *P* = 2.2e−16) (Fig. 4b, right). In contrast, there was only a weak relationship between the cumulative abundance of taxa characterizing cluster two, mainly composed of *Gorilla* spp., and the observed number of taxa seen among members of this group (*R*^2^ = 0.006, *P* = 0.043) (Fig. 4b, center). These analyses indicate that the abundance of taxa characterizing primates in cluster one could be associated with a gut environment conducive to increased diversity, contrary to the possible impact that cluster three-specific taxa could have.

## DISCUSSION

The results of our analyses highlight a level of microbiome plasticity in the human gut microbiome that challenges a paradigm of host phylogenetic constraints, as the primary driver of gut microbiome composition among primates (6). Instead, it is likely that gut microbial communities across different human populations and other primates are stimulated by diet, to the point phylogenetic signals are overridden across genetically distant primates sharing generalist or omnivorous dietary behaviors. As such, the simplified typology that assumes that host selective pressures shape the primate gut microbiome is not demonstrated in the data presented.

Although host phylogenetic constraints on the gut microbiome of primates have been documented to the extent of overriding dietary drivers (7), the expanded comparative approach utilized here shows significant gut microbiome overlap between humans and phylogenetically distant wild primates. Specifically, given the more traditional subsistence strategies associated with hunting and gathering or traditional agriculture, the data show that human gut microbiomes converge, to a large extent, with those of certain species of Old World monkeys from the *Cercopithecidae* family rather than with the phylogenetically closer African apes. Thus, given the wide molecular divergence between hominoids and cercopithecines, these data support an ecological model rather than evolutionary convergence on primate gut microbiomes.

### Ecological similarities between cercopithecines and human populations under traditional lifestyles.

Old World monkeys such as mangabeys, baboons, and vervets, which showed the greatest overlap with the gut microbiome of the BaAka hunter-gatherers and Bantu traditional agriculturists, maintain a more diverse and eclectic diet than the relatively more specialized diet of wild great apes, who concentrate on ripe fruit or highly nutritional vegetation when seasonally available (22–24). In contrast, the diets of olive baboons, mangabeys, and vervets is comprised of a remarkable range of fruits, seeds, flowers, shrubs, herbaceous plants, tubers, tree gums, grasses, sedge corms, insects, eggs, and even small vertebrates (25). Increased dietary diversity in these primates serves to broaden a more complex nutritional profile than that of sympatric primates, such as gorillas or chimpanzees. Hence, it is likely that gut microbiome adaptations to dietary complexity allow cercopithecines to exploit foraging resources with wide variation in sugars, fiber, protein, lipids, minerals, and secondary compounds, being less selective in their dietary choices and relying on a great variety of nutrient fractions, even within a single category of foods (e.g., different fruits and fruit parts) (22, 23).

Along these lines, the microbiome convergence reported here is particularly surprising, given that cooking among all human groups should, theoretically, dampen or even negate among-group microbiome differences. However, beyond cooking, dietary and nutritional variety is also a defining subsistence trait in nonindustrialized human populations, especially in contemporary hunter-gatherers (3, 4). For instance, BaAka hunter-gatherers rely on a wide variety of foods in their diet, including fruits, nuts, mushrooms, grains, leaves, wild game, insects, fish, and both wild and cultivated tubers (26). Although the Bantu agriculturalists rely to a greater degree on a market-based subsistence strategy, they still exhibit considerable overlap in dietary behaviors with the BaAka communities, particularly in their reliance on tubers. This trait is reflected in the gut microbiome of both groups, unlike that of subjects from industrialized societies (1). Thus, the high microbiome similarity observed between nonindustrial human populations and certain Old World monkeys may reflect similar reliance on a wide variety of wild food items with similarly complex nutritional profiles. Among these dietary components, starches, hexoses, cellulose, hemicellulose, and pectic compounds, all characteristics of wild primate diets, may have a role in driving these similar microbiome traits (27). However, the specific dietary behaviors and nutritional fractions driving this convergence remain unclear.

### Common microbiome signatures in cercopithecines and human populations under traditional subsistence patterns.

Some of the genera that characterized microbiome convergence between humans following traditional lifestyles and cercopithecines (Fig. 3a) (e.g., *Coprococcus*, *Clostridium*, *Faecalibacterium*, and *Lachnospira*) are typically associated with the dietary inclusion of fermentable fibers, principally in the form of starch and pectin-rich substrates (28, 29). As such, reliance on starchy fruits, legumes, seeds, grains, and tubers, all sources of starch (30), may be common drivers of the cercopithecine-human microbiome convergence reported here. However, the impact of other diverse dietary substrates, such as fats, proteins, minerals, vitamins, and phenolics, also should be considered in explaining this convergence (31).

It should be noted that one of the most distinctive taxonomic characteristics detected among members of the cluster primarily composed of nonindustrialized humans and the cercopithecines is the abundance of *Prevotella*, which has been consistently associated with nonwestern, small-scale rural and hunter-gatherer populations in Africa, South America, Asia, and the South Pacific (1, 2, 10, 32–34). *Prevotella* has also been reported to define an enterotype of western subjects consuming plant-based diets (35). However, we and others have also reported that, in nonhuman primates (apes and Old World monkeys), abundances of *Prevotella* increase when there is consumption of more digestible carbohydrate sources, during high-fruit seasons (13), in captivity (15), or in controlled experiments that evaluate the effect of western diets on their gut microbiome (36). Also, we report that abundances of *Prevotella* are lowest not only among U.S. humans and captive capuchins (cluster three) but also among primates consuming highly fibrous diets (*Gorilla* spp. in cluster two). Moreover, the group of western researchers temporarily switching to a traditional lifestyle in the field still showed abundances of *Prevotella* that are comparable to those seen in cercopithecine monkeys (see Fig. S6 in the supplemental material).

10.1128/mSphere.00271-19.6 FIG S6 Relative abundance of *Prevotella* in the fecal microbiome of different primate species. The dotted box is drawn to enclose groups where the medians are not statistically different (*P* > 0.05) according to Kruskal-Wallis multiple comparisons. Lower side bars (*x* axis) indicate cluster membership. Download FIG S6, TIFF file, 2.3 MB. Copyright © 2019 Gomez et al. 2019 Gomez et al. This content is distributed under the terms of the Creative Commons Attribution 4.0 International license.

Thus, abundances of *Prevotella* in humans and nonhuman primates may not correspond to consumption of hard-to-digest complex fibers, like those characterizing leaves or herbaceous vegetation, but with consumption of more fermentable, still complex carbohydrates and other chemically diverse substrates. In human populations under traditional subsistence lifestyles, both wild and cultivated tubers, legumes, maize, nuts, and grains are important staple foods and sources of fermentable starches (34, 37, 38). Thus, investigating the specific drivers of the abundance of *Prevotella* in the gut microbiome of distant primate species (humans and cercopithecines) requires a comprehensive nutritional analysis of common foods in tandem with detailed dietary assessments and functional microbiome surveys.

An important finding of this study was the observation that the greatest microbiome diversity links certain Old World monkeys and human populations practicing traditional subsistence, relative to primates that rely on diets with the highest content of complex fibers (e.g., gorillas). Although dietary fiber deprivation has been recognized as a critical factor triggering depletion of diversity in the human gut microbiome (39, 40), not all fibers are nutritionally or digestively equal, and not all dietary substrates that escape digestion in the proximal gut can stimulate microbial expansion in the distal gut. Hence, based on these results, it may be necessary to reconsider the set of factors that generate enriched diversity of gut microbiomes in populations following nonindustrialized lifestyles, in contrast to humans consuming western diets or folivorous primates. For example, microbial diversity may also be associated with substrates that are easily accessible by gut microbes in the distal gut, such as starches and other soluble fibers, rather than with the consumption of lignified, complex polysaccharides (39). Likewise, these results show that the presence of traditional microbes, largely absent from western humans and captive capuchins, is associated with increased diversity (Fig. 4d), a phenomenon possibly linked to increased substrate availability and critical cross-feeding reactions integrated in metabolic networks (41, 42) and facilitated by taxa such as *Prevotella*.

### Implications for understanding the human gut microbiome.

These results have implications for understanding the evolution of the human microbiome and its current configurations in the context of industrialized lifestyles, health, and disease. For example, traditional populations have been proposed as a model to understand human evolution in light of optimal health traits (43–45). As such, identifying the nutritional components of their diets and those of wild cercopithecines may help us determine whether more diverse fermentable polysaccharides leads to microbiome configurations that are different from those seen in western populations.

The results presented here also open the question of a possible discordance between humans and their gut microbiomes, especially for populations following industrialized subsistence strategies. Such (evolutionary) discordance has been proposed for human diets, where departures from traditional subsistence patterns and the adoption of modern diets are hypothesized to have rendered human genomes less adapted to rapid dietary changes along with industrialization (43, 46). This genome-diet mismatch hypothesis proposes that our digestive systems are maladapted to certain characteristics of industrialized diets, such as foods with high glycemic loads and high levels of processed fatty acids, altered macro- and micronutrient profiles, and lower fiber content. These dietary characteristics are believed to be associated with the high incidence of chronic diseases observed in western societies (e.g., diabetes, cardiovascular disease, and cancer, among others).

Thus, an important goal lies in determining the extent to which our second genome, the gut microbiome, has also been associated with this mismatch, along with departures from traditional subsistence and following industrialization. This association has been demonstrated recently, showing that human populations under traditional lifestyles experience significant alterations in their gut microbiomes and health after adopting a westernized lifestyle (32). Furthermore, whether such microbiome discordance can be reversed is unclear. In this regard, while it has recently been shown that immersion of 5 urban subjects in traditional lifestyles and diets of rainforest villagers for 16 days did not modify their microbiomes (47), we show that 5 western researchers switching to traditional lifestyles in the field, from 3 to 6 months, acquired microbiome configurations similar to those of the traditional populations they lived with, deviating significantly from the microbiomes of U.S. subjects (HMP).

Thus, the plasticity of the human microbiome presented here, in the context of degree and time of exposure to nonindustrialized lifestyles, raises questions about the potential of dietary manipulations that can reset the microbiome in populations under westernized subsistence patterns. However, given the long-term exposure of the human microbiome to industrialized diets since the agricultural and industrial revolutions, it is unclear whether strategies to reset or change gut microbiome configurations in industrialized populations should only include dietary manipulations or both dietary and environmental microbial exposures. This issue makes the premise of microbiome-based therapeutic strategies to improve human health more challenging.

### Conclusions.

In summary, the expanded comparative approach presented here indicates that subsistence patterns, such as those exhibited by contemporary hunter-gatherers or traditional agriculturalists, are associated with gut microbiome composition and diversity characterizing distantly related primates that exploit a broad-based diet. Although we largely rely on data collected by our group (except for the HMP data set), limitations related to sample sizes in the cercopithecine groups analyzed, and lack of specific diet composition and feeding behavioral data, warrant careful consideration of these results. We encourage future analyses to strengthen and validate the presented results and the use of functional approaches to uncover gene-centric and strain-level gut microbiome similarities among larger cohorts of cercopithecines and different human populations.

Characterizing host-microbe interactions at the functional level in the gut of human populations, and nonhuman primates with various degrees of subsistence, also should provide the foundation to further understand a potential discordance between human genomes, their gut microbiomes, and diets, in the context of westernized lifestyles and modern human diseases. More importantly, the specific drivers of the human-cercopithecine microbiome convergence reported here remain unknown and should be studied rigorously. Specifically, the nutritional fractions triggering the gut microbiome convergence between these distantly related primates need to be identified. This strategy may help in developing potential applications of traditional dietary interventions to improve human health through modulating the gut microbiome.

## MATERIALS AND METHODS

### Subjects and samples.

Samples from western lowland gorillas (Gorilla gorilla *gorilla*, *n* = 191), Central African chimpanzees (Pan troglodytes *troglodytes*, *n* = 10), agile mangabeys (Cercocebus agilis, *n* = 10), BaAka hunter-gatherers (*n* = 28), Bantu agriculturalists (*n* = 29), and western researchers (*n* = 5) were collected at the Dzanga Sangha Protected Areas, Central African Republic, as described in Gomez et al. (1, 13). Samples from mountain gorillas (Gorilla beringei *beringei*, *n* = 48) were collected at Bwindi Impenetrable National Park, Uganda, also as described in Gomez et al. (13). Samples from vervets (Chlorocebus aethiops, *n* = 23) were collected at the island of St. Kitts as described in Amato et al. (36), and samples from black howlers (Alouatta pigra, *n* = 33) were collected at Palenque National Park, Mexico, as described in Amato et al. (48). Samples from olive baboons (Papio anubis, *n* = 4) were collected at Awash National Park, Ethiopia, samples from geladas (Theropithecus gelada, *n* = 7) at Guassa Plateau, Ethiopia, and samples from captive tufted capuchins (Cebus apella, *n* = 4) at the National Institutes of Health. Briefly, all samples were collected in RNAlater (2 volumes of solution × 1 g of fecal sample) for a maximum of 1 month in the field before storage at −80 or −20°C until processing, except samples from tufted capuchins, which were frozen at −80°C right after collection. 16S rRNA sequences of U.S. subjects (*n* = 56), part of the Human Microbiome Project (HMP) (18, 19), were downloaded from https://hmpdacc.org/.

### 16S rRNA gene analyses.

All sequences used in this study were generated using the same 16S rRNA variable region, the same sequencing platform, and the same DNA extraction kit. After extraction of genomic DNA using the Power Soil DNA extraction kit of MoBio (Carlsbad, CA), the 16S rRNA gene was sequenced targeting the V1-V3 variable region. PCR (25 cycles of 94°C for 30 s, 50°C for 30 s, and 72°C for 45 s) used barcoded primers (27F, 5′-AGAGTTTGATYMTGGCTCAG-3′; 534R, 5′-ATTACCGCGGCTGCTGG-3′) on the Genome Sequencer FLX with GS FLX Titanium series at the J. Craig Venter Institute (Rockville, MD) as described previously (1, 48). The analyses of 16S rRNA sequence data were conducted using custom-made Perl scripts and the QIIME pipeline (v1.9) (49). Briefly, Perl scripts were used to locate primer sequences (27f and 535r), separate forward and reverse reads, reverse complement the reverse reads, and remove duplicates, keeping reads from 200 nucleotides (nt) to a maximum of 535 nt and a maximum number of homopolymers of 6. Processed sequences were used to pick operational taxonomic units (OTUs) based on an open-reference approach with a threshold of 97% identity to the Greengenes database (v13_8). However, most analyses, except for alpha diversity determinations, were conducted at the genus level after collapsing reads at different taxonomic levels using the summarize_taxa.py function in QIIME.

### Statistical analyses.

All microbial community ecology analyses were performed within the R statistical interface (50). Briefly, all ordination analyses (principal coordinate analysis) and distance matrices (Bray, UniFrac, and Canberra) were performed using the R phyloseq package (51). Alpha diversity estimates, permutational multivariate analyses of variance (PERMANOVA), and partitioning around medoids (PAM clustering) were calculated using the R vegan package (52). RandomForest classification (500 trees) as implemented in the randomForest R package (53) was used to corroborate cluster membership. The mean decrease in abundance index (>9) of randomForest in combination with false discovery rate (FDR)-adjusted Kruskal-Wallis multiple comparisons (*q* < 0.05) and species indicator analysis (indicator values, >0.5; *P* < 0.05), as implemented in the labdsv R package (54), were used to detect taxa differentially abundant in each cluster. An unrooted tree based on 16S rRNA Bray-Curtis distances was performed using average genus abundances for each primate group and the hclust function within the R ape package (55). An unrooted phylogenetic tree based on mtDNA sequences was also generated from representative data downloaded from https://www.ncbi.nlm.nih.gov/genomes/GenomesGroup.cgi?opt=organelle&taxid=2759, using ClustalW-based multiple-sequence alignments and plotted with the ggtree package in R (56, 57). All graphs were made using the ggplots R package (58).

### Data availability.

The accession numbers for 16S rRNA sequence data are the following: data for the BaAka and Bantu peoples are deposited in project MG-RAST under 16608 (1); sequence data from western lowland and mountain gorillas are deposited in project MG-RAST under mgp6321 and mgp13961 (13); data from vervets and agile mangabeys are in the NCBI Sequence Read Archive (SRA) under SRP065516 (36). Data from howlers (48), baboons, geladas, capuchins, chimpanzees, and western researchers working in the Dzanga Sangha Protected Areas, Central African Republic African field site, are deposited in MG-RAST under mgp89894.
