# Erratum for Gomez et al., “Plasticity in the Human Gut Microbiome Defies Evolutionary Constraints”

**DOI:** 10.1128/mSphere.00168-21

**Published:** 2021-03-17

**Authors:** Andres Gomez, Ashok Kumar Sharma, Elizabeth K. Mallott, Klara J. Petrzelkova, Carolyn A. Jost Robinson, Carl J. Yeoman, Franck Carbonero, Barbora Pafco, Jessica M. Rothman, Alexander Ulanov, Klara Vlckova, Katherine R. Amato, Stephanie L. Schnorr, Nathaniel J. Dominy, David Modry, Angelique Todd, Manolito Torralba, Karen E. Nelson, Michael B. Burns, Ran Blekhman, Melissa Remis, Rebecca M. Stumpf, Brenda A. Wilson, H. Rex Gaskins, Paul A. Garber, Bryan A. White, Steven R. Leigh

**Affiliations:** a Department of Animal Science, University of Minnesota, Twin Cities, St. Paul, Minnesota, USA; b Department of Anthropology, Northwestern University, Evanston, Illinois, USA; c Institute of Vertebrate Biology, The Czech Academy of Sciences, Brno, Czech Republic; d Institute of Parasitology, Biology Centre of the Czech Academy of Sciences, Ceske Budejovice, Czech Republic; e Liberec Zoo, Liberec, Czech Republic; f Department of Anthropology, University of North Carolina, Wilmington, North Carolina, USA; g Department of Animal and Range Sciences, Montana State University, Bozeman, Montana, USA; h Department of Nutrition & Exercise Physiology, Elson S. Floyd College of Medicine, Washington State University, Spokane, Washington, USA; i Department of Pathology and Parasitology, Faculty of Veterinary Medicine, University of Veterinary and Pharmaceutical Sciences Brno, Brno, Czech Republic; j Department of Anthropology, Hunter College of CUNY and New York Consortium in Evolutionary Primatology (NYCEP), New York, New York, USA; k Metabolomics Center, Roy J. Carver Biotechnology Center, University of Illinois at Urbana-Champaign, Champaign, Illinois, USA; l Department of Anthropology, University of Nevada, Las Vegas, Nevada, USA; m Department of Anthropology, Dartmouth College, Hanover, New Hampshire, USA; n Central European Institute for Technology (CEITEC), University of Veterinary and Pharmaceutical Sciences Brno, Brno, Czech Republic; o World Wildlife Fund, Dzanga-Sangha Protected Areas, Bayanga, Central African Republic; p J. Craig Venter Institute, La Jolla, California, USA; q Department of Biology, Loyola University Chicago, Chicago, Illinois, USA; r Department of Genetics, Cell Biology, and Development, University of Minnesota, Twin Cities, Minneapolis, Minnesota, USA; s Department of Anthropology, Purdue University, West Lafayette, Indiana, USA; t Carl Woese Institute for Genomic Biology, University of Illinois at Urbana-Champaign, Champaign, Illinois, USA; u Department of Anthropology, University of Illinois at Urbana-Champaign, Champaign, Illinois, USA; v Department of Microbiology, University of Illinois at Urbana-Champaign, Champaign, Illinois, USA; w Department of Animal Sciences, University of Illinois at Urbana-Champaign, Champaign, Illinois, USA; x Department of Anthropology, University of Colorado, Boulder, Colorado, USA; y Konrad Lorenz Institute for Evolution and Cognition Research, Klosterneuburg, Austria

## ERRATUM

Volume 4, no. 4, e00271-19, 2019, https://doi.org/10.1128/mSphere.00271-19. The following sentence should be added to the end of the first paragraph of the Acknowledgments section: “Collection and export of fecal samples was also facilitated by the Filoha Hamadryas Project with the generous permission of the Ethiopian Wildlife Conservation Authority.”

