# Supplementary material for: Plasticity in the Human Gut Microbiome Defies Evolutionary Constraints
Source: mSphere. 2019 Jul 31;4(4):e00271-19. doi: 10.1128/mSphere.00271-19 (PMC6669335; doi:10.1128/mSphere.00271-19)
Supplement: TABLE S1 [file mSphere.00271-19-st001.docx]

| **Sample** | **Sequencing depth** | **Common name** | ***Scientific name*** | **Origin** |
| --- | --- | --- | --- | --- |
| M.100 | 6179 | Agile Mangabey | *Cercocebus agilis* | Dzangha Sangha, Central African Republic |
| M.157 | 5568 | Agile Mangabey | *Cercocebus agilis* | Dzangha Sangha, Central African Republic |
| M.158 | 3265 | Agile Mangabey | *Cercocebus agilis* | Dzangha Sangha, Central African Republic |
| M.159 | 3521 | Agile Mangabey | *Cercocebus agilis* | Dzangha Sangha, Central African Republic |
| M.160 | 4247 | Agile Mangabey | *Cercocebus agilis* | Dzangha Sangha, Central African Republic |
| M.252 | 4032 | Agile Mangabey | *Cercocebus agilis* | Dzangha Sangha, Central African Republic |
| M.45 | 3385 | Agile Mangabey | *Cercocebus agilis* | Dzangha Sangha, Central African Republic |
| M.47 | 8968 | Agile Mangabey | *Cercocebus agilis* | Dzangha Sangha, Central African Republic |
| M.48 | 5055 | Agile Mangabey | *Cercocebus agilis* | Dzangha Sangha, Central African Republic |
| M.51 | 4305 | Agile Mangabey | *Cercocebus agilis* | Dzangha Sangha, Central African Republic |
| Hu.107 | 14098 | BaAka Hunter Gatherer | *Homo sapiens* | Dzangha Sangha, Central African Republic |
| Hu.112 | 12972 | BaAka Hunter Gatherer | *Homo sapiens* | Dzangha Sangha, Central African Republic |
| Hu.113 | 16112 | BaAka Hunter Gatherer | *Homo sapiens* | Dzangha Sangha, Central African Republic |
| Hu.117 | 12348 | BaAka Hunter Gatherer | *Homo sapiens* | Dzangha Sangha, Central African Republic |
| Hu.118 | 12880 | BaAka Hunter Gatherer | *Homo sapiens* | Dzangha Sangha, Central African Republic |
| Hu.122 | 8010 | BaAka Hunter Gatherer | *Homo sapiens* | Dzangha Sangha, Central African Republic |
| Hu.123 | 12291 | BaAka Hunter Gatherer | *Homo sapiens* | Dzangha Sangha, Central African Republic |
| Hu.500 | 6471 | BaAka Hunter Gatherer | *Homo sapiens* | Dzangha Sangha, Central African Republic |
| Hu.501 | 9431 | BaAka Hunter Gatherer | *Homo sapiens* | Dzangha Sangha, Central African Republic |
| Hu.503 | 6774 | BaAka Hunter Gatherer | *Homo sapiens* | Dzangha Sangha, Central African Republic |
| Hu.506 | 9562 | BaAka Hunter Gatherer | *Homo sapiens* | Dzangha Sangha, Central African Republic |
| Hu.521 | 6592 | BaAka Hunter Gatherer | *Homo sapiens* | Dzangha Sangha, Central African Republic |
| Hu.522 | 5373 | BaAka Hunter Gatherer | *Homo sapiens* | Dzangha Sangha, Central African Republic |
| Hu.523 | 4595 | BaAka Hunter Gatherer | *Homo sapiens* | Dzangha Sangha, Central African Republic |
| Hu.524 | 4935 | BaAka Hunter Gatherer | *Homo sapiens* | Dzangha Sangha, Central African Republic |
| Hu.525 | 6734 | BaAka Hunter Gatherer | *Homo sapiens* | Dzangha Sangha, Central African Republic |
| Hu.526 | 5360 | BaAka Hunter Gatherer | *Homo sapiens* | Dzangha Sangha, Central African Republic |
| Hu.527 | 4634 | BaAka Hunter Gatherer | *Homo sapiens* | Dzangha Sangha, Central African Republic |
| Hu.529 | 4426 | BaAka Hunter Gatherer | *Homo sapiens* | Dzangha Sangha, Central African Republic |
| Hu.530 | 7986 | BaAka Hunter Gatherer | *Homo sapiens* | Dzangha Sangha, Central African Republic |
| Hu.531 | 6825 | BaAka Hunter Gatherer | *Homo sapiens* | Dzangha Sangha, Central African Republic |
| Hu.532 | 3626 | BaAka Hunter Gatherer | *Homo sapiens* | Dzangha Sangha, Central African Republic |
| Hu.534 | 6239 | BaAka Hunter Gatherer | *Homo sapiens* | Dzangha Sangha, Central African Republic |
| Hu.536 | 6007 | BaAka Hunter Gatherer | *Homo sapiens* | Dzangha Sangha, Central African Republic |
| Hu.537 | 4449 | BaAka Hunter Gatherer | *Homo sapiens* | Dzangha Sangha, Central African Republic |
| Hu.538 | 4308 | BaAka Hunter Gatherer | *Homo sapiens* | Dzangha Sangha, Central African Republic |
| Hu.539 | 5083 | BaAka Hunter Gatherer | *Homo sapiens* | Dzangha Sangha, Central African Republic |
| Hu.540 | 9515 | BaAka Hunter Gatherer | *Homo sapiens* | Dzangha Sangha, Central African Republic |
| Hu.100 | 12815 | Bantu Agriculturalist | *Homo sapiens* | Dzangha Sangha, Central African Republic |
| Hu.101 | 10179 | Bantu Agriculturalist | *Homo sapiens* | Dzangha Sangha, Central African Republic |
| Hu.102 | 10580 | Bantu Agriculturalist | *Homo sapiens* | Dzangha Sangha, Central African Republic |
| Hu.103 | 4889 | Bantu Agriculturalist | *Homo sapiens* | Dzangha Sangha, Central African Republic |
| Hu.104 | 10407 | Bantu Agriculturalist | *Homo sapiens* | Dzangha Sangha, Central African Republic |
| Hu.105 | 13807 | Bantu Agriculturalist | *Homo sapiens* | Dzangha Sangha, Central African Republic |
| Hu.106 | 12622 | Bantu Agriculturalist | *Homo sapiens* | Dzangha Sangha, Central African Republic |
| Hu.108 | 11855 | Bantu Agriculturalist | *Homo sapiens* | Dzangha Sangha, Central African Republic |
| Hu.109 | 15744 | Bantu Agriculturalist | *Homo sapiens* | Dzangha Sangha, Central African Republic |
| Hu.110 | 12783 | Bantu Agriculturalist | *Homo sapiens* | Dzangha Sangha, Central African Republic |
| Hu.111 | 16015 | Bantu Agriculturalist | *Homo sapiens* | Dzangha Sangha, Central African Republic |
| Hu.114 | 9524 | Bantu Agriculturalist | *Homo sapiens* | Dzangha Sangha, Central African Republic |
| Hu.115 | 9851 | Bantu Agriculturalist | *Homo sapiens* | Dzangha Sangha, Central African Republic |
| Hu.116 | 11893 | Bantu Agriculturalist | *Homo sapiens* | Dzangha Sangha, Central African Republic |
| Hu.119 | 12409 | Bantu Agriculturalist | *Homo sapiens* | Dzangha Sangha, Central African Republic |
| Hu.120 | 9483 | Bantu Agriculturalist | *Homo sapiens* | Dzangha Sangha, Central African Republic |
| Hu.121 | 16669 | Bantu Agriculturalist | *Homo sapiens* | Dzangha Sangha, Central African Republic |
| Hu.124 | 11887 | Bantu Agriculturalist | *Homo sapiens* | Dzangha Sangha, Central African Republic |
| Hu.502 | 8183 | Bantu Agriculturalist | *Homo sapiens* | Dzangha Sangha, Central African Republic |
| Hu.504 | 12091 | Bantu Agriculturalist | *Homo sapiens* | Dzangha Sangha, Central African Republic |
| Hu.505 | 6513 | Bantu Agriculturalist | *Homo sapiens* | Dzangha Sangha, Central African Republic |
| Hu.507 | 8549 | Bantu Agriculturalist | *Homo sapiens* | Dzangha Sangha, Central African Republic |
| Hu.508 | 8022 | Bantu Agriculturalist | *Homo sapiens* | Dzangha Sangha, Central African Republic |
| Hu.517 | 5806 | Bantu Agriculturalist | *Homo sapiens* | Dzangha Sangha, Central African Republic |
| Hu.518 | 5628 | Bantu Agriculturalist | *Homo sapiens* | Dzangha Sangha, Central African Republic |
| Hu.528 | 3563 | Bantu Agriculturalist | *Homo sapiens* | Dzangha Sangha, Central African Republic |
| Hu.533 | 4038 | Bantu Agriculturalist | *Homo sapiens* | Dzangha Sangha, Central African Republic |
| Hu.535 | 3310 | Bantu Agriculturalist | *Homo sapiens* | Dzangha Sangha, Central African Republic |
| Hu.99 | 16049 | Bantu Agriculturalist | *Homo sapiens* | Dzangha Sangha, Central African Republic |
| C.121 | 2732 | Central African Chimpanzee | *Pan troglodytes troglodytes* | Dzangha Sangha, Central African Republic |
| C.128 | 10257 | Central African Chimpanzee | *Pan troglodytes troglodytes* | Dzangha Sangha, Central African Republic |
| C.129 | 8360 | Central African Chimpanzee | *Pan troglodytes troglodytes* | Dzangha Sangha, Central African Republic |
| C.28 | 5570 | Central African Chimpanzee | *Pan troglodytes troglodytes* | Dzangha Sangha, Central African Republic |
| C.29 | 13354 | Central African Chimpanzee | *Pan troglodytes troglodytes* | Dzangha Sangha, Central African Republic |
| C.30 | 8300 | Central African Chimpanzee | *Pan troglodytes troglodytes* | Dzangha Sangha, Central African Republic |
| C.34 | 4050 | Central African Chimpanzee | *Pan troglodytes troglodytes* | Dzangha Sangha, Central African Republic |
| C.44 | 5122 | Central African Chimpanzee | *Pan troglodytes troglodytes* | Dzangha Sangha, Central African Republic |
| C.45 | 8428 | Central African Chimpanzee | *Pan troglodytes troglodytes* | Dzangha Sangha, Central African Republic |
| C.54 | 11237 | Central African Chimpanzee | *Pan troglodytes troglodytes* | Dzangha Sangha, Central African Republic |
| 1 | 10141 | Gelada | *Theropithecus gelada* | Guassa Plateau, Ethiopia |
| 2 | 10376 | Gelada | *Theropithecus gelada* | Guassa Plateau, Ethiopia |
| 3 | 7857 | Gelada | *Theropithecus gelada* | Guassa Plateau, Ethiopia |
| 4 | 10449 | Gelada | *Theropithecus gelada* | Guassa Plateau, Ethiopia |
| 6 | 12448 | Gelada | *Theropithecus gelada* | Guassa Plateau, Ethiopia |
| 7 | 10393 | Gelada | *Theropithecus gelada* | Guassa Plateau, Ethiopia |
| 8 | 8566 | Gelada | *Theropithecus gelada* | Guassa Plateau, Ethiopia |
| HMP1 | 4779 | Human (HMP) | *Homo sapiens* | USA |
| HMP10 | 6390 | Human (HMP) | *Homo sapiens* | USA |
| HMP12 | 8673 | Human (HMP) | *Homo sapiens* | USA |
| HMP14 | 4341 | Human (HMP) | *Homo sapiens* | USA |
| HMP15 | 1779 | Human (HMP) | *Homo sapiens* | USA |
| HMP16 | 10294 | Human (HMP) | *Homo sapiens* | USA |
| HMP17 | 3101 | Human (HMP) | *Homo sapiens* | USA |
| HMP18 | 9069 | Human (HMP) | *Homo sapiens* | USA |
| HMP19 | 1291 | Human (HMP) | *Homo sapiens* | USA |
| HMP2 | 9897 | Human (HMP) | *Homo sapiens* | USA |
| HMP20 | 4170 | Human (HMP) | *Homo sapiens* | USA |
| HMP21 | 8051 | Human (HMP) | *Homo sapiens* | USA |
| HMP22 | 1574 | Human (HMP) | *Homo sapiens* | USA |
| HMP23 | 2195 | Human (HMP) | *Homo sapiens* | USA |
| HMP24 | 6691 | Human (HMP) | *Homo sapiens* | USA |
| HMP25 | 3611 | Human (HMP) | *Homo sapiens* | USA |
| HMP26 | 10148 | Human (HMP) | *Homo sapiens* | USA |
| HMP27 | 2051 | Human (HMP) | *Homo sapiens* | USA |
| HMP28 | 17117 | Human (HMP) | *Homo sapiens* | USA |
| HMP29 | 3327 | Human (HMP) | *Homo sapiens* | USA |
| HMP3 | 8490 | Human (HMP) | *Homo sapiens* | USA |
| HMP30 | 2641 | Human (HMP) | *Homo sapiens* | USA |
| HMP31 | 13842 | Human (HMP) | *Homo sapiens* | USA |
| HMP32 | 6204 | Human (HMP) | *Homo sapiens* | USA |
| HMP33 | 4865 | Human (HMP) | *Homo sapiens* | USA |
| HMP35 | 7657 | Human (HMP) | *Homo sapiens* | USA |
| HMP36 | 4012 | Human (HMP) | *Homo sapiens* | USA |
| HMP39 | 3748 | Human (HMP) | *Homo sapiens* | USA |
| HMP4 | 7320 | Human (HMP) | *Homo sapiens* | USA |
| HMP40 | 3883 | Human (HMP) | *Homo sapiens* | USA |
| HMP41 | 3443 | Human (HMP) | *Homo sapiens* | USA |
| HMP42 | 1365 | Human (HMP) | *Homo sapiens* | USA |
| HMP43 | 1208 | Human (HMP) | *Homo sapiens* | USA |
| HMP44 | 4824 | Human (HMP) | *Homo sapiens* | USA |
| HMP45 | 4265 | Human (HMP) | *Homo sapiens* | USA |
| HMP46 | 4462 | Human (HMP) | *Homo sapiens* | USA |
| HMP47 | 3994 | Human (HMP) | *Homo sapiens* | USA |
| HMP48 | 4423 | Human (HMP) | *Homo sapiens* | USA |
| HMP49 | 3986 | Human (HMP) | *Homo sapiens* | USA |
| HMP5 | 4862 | Human (HMP) | *Homo sapiens* | USA |
| HMP50 | 3274 | Human (HMP) | *Homo sapiens* | USA |
| HMP51 | 2609 | Human (HMP) | *Homo sapiens* | USA |
| HMP52 | 4927 | Human (HMP) | *Homo sapiens* | USA |
| HMP53 | 4275 | Human (HMP) | *Homo sapiens* | USA |
| HMP54 | 4734 | Human (HMP) | *Homo sapiens* | USA |
| HMP55 | 4511 | Human (HMP) | *Homo sapiens* | USA |
| HMP56 | 3818 | Human (HMP) | *Homo sapiens* | USA |
| HMP57 | 3392 | Human (HMP) | *Homo sapiens* | USA |
| HMP58 | 5392 | Human (HMP) | *Homo sapiens* | USA |
| HMP59 | 4971 | Human (HMP) | *Homo sapiens* | USA |
| HMP6 | 4578 | Human (HMP) | *Homo sapiens* | USA |
| HMP60 | 4189 | Human (HMP) | *Homo sapiens* | USA |
| HMP61 | 3723 | Human (HMP) | *Homo sapiens* | USA |
| HMP7 | 2971 | Human (HMP) | *Homo sapiens* | USA |
| HMP8 | 3428 | Human (HMP) | *Homo sapiens* | USA |
| HMP9 | 1524 | Human (HMP) | *Homo sapiens* | USA |
| 13 | 8395 | Black Howler | *Alouatta pigra* | Palenque National Park, Mexico |
| 15 | 5120 | Black Howler | *Alouatta pigra* | Palenque National Park, Mexico |
| 16 | 9864 | Black Howler | *Alouatta pigra* | Palenque National Park, Mexico |
| 17 | 11375 | Black Howler | *Alouatta pigra* | Palenque National Park, Mexico |
| 18 | 4471 | Black Howler | *Alouatta pigra* | Palenque National Park, Mexico |
| 19 | 7204 | Black Howler | *Alouatta pigra* | Palenque National Park, Mexico |
| 24 | 12437 | Black Howler | *Alouatta pigra* | Palenque National Park, Mexico |
| 42 | 6403 | Black Howler | *Alouatta pigra* | Palenque National Park, Mexico |
| 49 | 22636 | Black Howler | *Alouatta pigra* | Palenque National Park, Mexico |
| 52 | 17284 | Black Howler | *Alouatta pigra* | Palenque National Park, Mexico |
| 55 | 10472 | Black Howler | *Alouatta pigra* | Palenque National Park, Mexico |
| 59 | 10016 | Black Howler | *Alouatta pigra* | Palenque National Park, Mexico |
| 60 | 18782 | Black Howler | *Alouatta pigra* | Palenque National Park, Mexico |
| 65 | 10583 | Black Howler | *Alouatta pigra* | Palenque National Park, Mexico |
| 103 | 3438 | Black Howler | *Alouatta pigra* | Palenque National Park, Mexico |
| 105 | 3517 | Black Howler | *Alouatta pigra* | Palenque National Park, Mexico |
| 107 | 4354 | Black Howler | *Alouatta pigra* | Palenque National Park, Mexico |
| 108 | 3323 | Black Howler | *Alouatta pigra* | Palenque National Park, Mexico |
| 110 | 4654 | Black Howler | *Alouatta pigra* | Palenque National Park, Mexico |
| 111 | 4505 | Black Howler | *Alouatta pigra* | Palenque National Park, Mexico |
| 112 | 3619 | Black Howler | *Alouatta pigra* | Palenque National Park, Mexico |
| 116 | 7063 | Black Howler | *Alouatta pigra* | Palenque National Park, Mexico |
| 170 | 2877 | Black Howler | *Alouatta pigra* | Palenque National Park, Mexico |
| 172 | 3344 | Black Howler | *Alouatta pigra* | Palenque National Park, Mexico |
| 173 | 2608 | Black Howler | *Alouatta pigra* | Palenque National Park, Mexico |
| 175 | 2843 | Black Howler | *Alouatta pigra* | Palenque National Park, Mexico |
| 177 | 2565 | Black Howler | *Alouatta pigra* | Palenque National Park, Mexico |
| 178 | 2314 | Black Howler | *Alouatta pigra* | Palenque National Park, Mexico |
| 179 | 3004 | Black Howler | *Alouatta pigra* | Palenque National Park, Mexico |
| 180 | 2240 | Black Howler | *Alouatta pigra* | Palenque National Park, Mexico |
| 182 | 2079 | Black Howler | *Alouatta pigra* | Palenque National Park, Mexico |
| 12b | 7738 | Black Howler | *Alouatta pigra* | Palenque National Park, Mexico |
| 20E | 4838 | Black Howler | *Alouatta pigra* | Palenque National Park, Mexico |
| MB1 | 6355 | Mountain Gorilla | *Gorilla beringei beringei* | Bwindi Impenetrable National park, Uganda |
| MB2 | 4322 | Mountain Gorilla | *Gorilla beringei beringei* | Bwindi Impenetrable National park, Uganda |
| MB3 | 6086 | Mountain Gorilla | *Gorilla beringei beringei* | Bwindi Impenetrable National park, Uganda |
| MB4 | 5189 | Mountain Gorilla | *Gorilla beringei beringei* | Bwindi Impenetrable National Park, Uganda |
| MDF | 11049 | Mountain Gorilla | *Gorilla beringei beringei* | Bwindi Impenetrable National Park, Uganda |
| MH1 | 2906 | Mountain Gorilla | *Gorilla beringei beringei* | Bwindi Impenetrable National Park, Uganda |
| MH10 | 5542 | Mountain Gorilla | *Gorilla beringei beringei* | Bwindi Impenetrable National Park, Uganda |
| MH2 | 2274 | Mountain Gorilla | *Gorilla beringei beringei* | Bwindi Impenetrable National Park, Uganda |
| MH3 | 2754 | Mountain Gorilla | *Gorilla beringei beringei* | Bwindi Impenetrable National Park, Uganda |
| MH4 | 3062 | Mountain Gorilla | *Gorilla beringei beringei* | Bwindi Impenetrable National Park, Uganda |
| MH5 | 2451 | Mountain Gorilla | *Gorilla beringei beringei* | Bwindi Impenetrable National Park, Uganda |
| MH6 | 5414 | Mountain Gorilla | *Gorilla beringei beringei* | Bwindi Impenetrable National Park, Uganda |
| MH7 | 2180 | Mountain Gorilla | *Gorilla beringei beringei* | Bwindi Impenetrable National Park, Uganda |
| MH8 | 5614 | Mountain Gorilla | *Gorilla beringei beringei* | Bwindi Impenetrable National Park, Uganda |
| MH9 | 2829 | Mountain Gorilla | *Gorilla beringei beringei* | Bwindi Impenetrable National Park, Uganda |
| MK1 | 3279 | Mountain Gorilla | *Gorilla beringei beringei* | Bwindi Impenetrable National Park, Uganda |
| MK2 | 6272 | Mountain Gorilla | *Gorilla beringei beringei* | Bwindi Impenetrable National Park, Uganda |
| MK3 | 5460 | Mountain Gorilla | *Gorilla beringei beringei* | Bwindi Impenetrable National Park, Uganda |
| MK4 | 4255 | Mountain Gorilla | *Gorilla beringei beringei* | Bwindi Impenetrable National Park, Uganda |
| MK5 | 5975 | Mountain Gorilla | *Gorilla beringei beringei* | Bwindi Impenetrable National Park, Uganda |
| MK6 | 6861 | Mountain Gorilla | *Gorilla beringei beringei* | Bwindi Impenetrable National Park, Uganda |
| MK7 | 6038 | Mountain Gorilla | *Gorilla beringei beringei* | Bwindi Impenetrable National Park, Uganda |
| MK8 | 5447 | Mountain Gorilla | *Gorilla beringei beringei* | Bwindi Impenetrable National Park, Uganda |
| MM1 | 5427 | Mountain Gorilla | *Gorilla beringei beringei* | Bwindi Impenetrable National Park, Uganda |
| MM2 | 4187 | Mountain Gorilla | *Gorilla beringei beringei* | Bwindi Impenetrable National Park, Uganda |
| MM3 | 6300 | Mountain Gorilla | *Gorilla beringei beringei* | Bwindi Impenetrable National Park, Uganda |
| MM4 | 3134 | Mountain Gorilla | *Gorilla beringei beringei* | Bwindi Impenetrable National Park, Uganda |
| MM5 | 4365 | Mountain Gorilla | *Gorilla beringei beringei* | Bwindi Impenetrable National Park, Uganda |
| MNK2 | 6186 | Mountain Gorilla | *Gorilla beringei beringei* | Bwindi Impenetrable National Park, Uganda |
| MNK3 | 5502 | Mountain Gorilla | *Gorilla beringei beringei* | Bwindi Impenetrable National Park, Uganda |
| MNK4 | 4000 | Mountain Gorilla | *Gorilla beringei beringei* | Bwindi Impenetrable National Park, Uganda |
| MNK5 | 3901 | Mountain Gorilla | *Gorilla beringei beringei* | Bwindi Impenetrable National Park, Uganda |
| MNK6 | 5667 | Mountain Gorilla | *Gorilla beringei beringei* | Bwindi Impenetrable National Park, Uganda |
| MR1 | 3796 | Mountain Gorilla | *Gorilla beringei beringei* | Bwindi Impenetrable National Park, Uganda |
| MR2 | 3028 | Mountain Gorilla | *Gorilla beringei beringei* | Bwindi Impenetrable National Park, Uganda |
| MR3 | 5806 | Mountain Gorilla | *Gorilla beringei beringei* | Bwindi Impenetrable National Park, Uganda |
| MR4 | 6511 | Mountain Gorilla | *Gorilla beringei beringei* | Bwindi Impenetrable National Park, Uganda |
| MR5 | 8733 | Mountain Gorilla | *Gorilla beringei beringei* | Bwindi Impenetrable National Park, Uganda |
| MR6 | 4052 | Mountain Gorilla | *Gorilla beringei beringei* | Bwindi Impenetrable National Park, Uganda |
| MR7 | 2356 | Mountain Gorilla | *Gorilla beringei beringei* | Bwindi Impenetrable National Park, Uganda |
| MR8 | 5453 | Mountain Gorilla | *Gorilla beringei beringei* | Bwindi Impenetrable National Park, Uganda |
| MS1 | 3160 | Mountain Gorilla | *Gorilla beringei beringei* | Bwindi Impenetrable National Park, Uganda |
| MS2 | 5792 | Mountain Gorilla | *Gorilla beringei beringei* | Bwindi Impenetrable National Park, Uganda |
| MS3 | 3754 | Mountain Gorilla | *Gorilla beringei beringei* | Bwindi Impenetrable National Park, Uganda |
| MS4 | 4501 | Mountain Gorilla | *Gorilla beringei beringei* | Bwindi Impenetrable National Park, Uganda |
| MS5 | 4405 | Mountain Gorilla | *Gorilla beringei beringei* | Bwindi Impenetrable National Park, Uganda |
| MS6 | 3575 | Mountain Gorilla | *Gorilla beringei beringei* | Bwindi Impenetrable National Park, Uganda |
| MS7 | 6197 | Mountain Gorilla | *Gorilla beringei beringei* | Bwindi Impenetrable National Park, Uganda |
| 9 | 8705 | Olive baboon | *Papio anubis* | Awash National Park, Ethiopia |
| 10 | 8853 | Olive baboon | *Papio anubis* | Awash National Park, Ethiopia |
| 11 | 15453 | Olive baboon | *Papio anubis* | Awash National Park, Ethiopia |
| 12a | 19837 | Olive baboon | *Papio anubis* | Awash National Park, Ethiopia |
| S1 | 15155 | Tufted Capuchin | *Cebus apella* | National Institute of Health, USA |
| S2 | 15195 | Tufted Capuchin | *Cebus apella* | National Institute of Health, USA |
| S3 | 16044 | Tufted Capuchin | *Cebus apella* | National Institute of Health, USA |
| S4 | 14600 | Tufted Capuchin | *Cebus apella* | National Institute of Health, USA |
| Vervet11 | 8971 | Vervet | *Chlorocebus aethiops* | St. Kitts |
| Vervet18 | 1344 | Vervet | *Chlorocebus aethiops* | St. Kitts |
| Vervet19 | 4368 | Vervet | *Chlorocebus aethiops* | St. Kitts |
| Vervet20 | 1902 | Vervet | *Chlorocebus aethiops* | St. Kitts |
| Vervet24 | 2145 | Vervet | *Chlorocebus aethiops* | St. Kitts |
| Vervet25 | 1948 | Vervet | *Chlorocebus aethiops* | St. Kitts |
| Vervet26 | 9215 | Vervet | *Chlorocebus aethiops* | St. Kitts |
| Vervet27 | 7487 | Vervet | *Chlorocebus aethiops* | St. Kitts |
| Vervet28 | 5457 | Vervet | *Chlorocebus aethiops* | St. Kitts |
| Vervet30 | 1999 | Vervet | *Chlorocebus aethiops* | St. Kitts |
| Vervet42 | 1693 | Vervet | *Chlorocebus aethiops* | St. Kitts |
| Vervet46 | 1238 | Vervet | *Chlorocebus aethiops* | St. Kitts |
| Vervet50 | 2108 | Vervet | *Chlorocebus aethiops* | St. Kitts |
| Vervet52 | 6596 | Vervet | *Chlorocebus aethiops* | St. Kitts |
| Vervet56 | 6963 | Vervet | *Chlorocebus aethiops* | St. Kitts |
| Vervet57 | 6017 | Vervet | *Chlorocebus aethiops* | St. Kitts |
| Vervet59 | 3927 | Vervet | *Chlorocebus aethiops* | St. Kitts |
| Vervet60 | 7980 | Vervet | *Chlorocebus aethiops* | St. Kitts |
| Vervet61 | 7155 | Vervet | *Chlorocebus aethiops* | St. Kitts |
| Vervet62 | 5279 | Vervet | *Chlorocebus aethiops* | St. Kitts |
| Vervet7 | 5119 | Vervet | *Chlorocebus aethiops* | St. Kitts |
| Vervet8 | 6256 | Vervet | *Chlorocebus aethiops* | St. Kitts |
| VervetW640 | 10808 | Vervet | *Chlorocebus aethiops* | St. Kitts |
| 2.2009 | 5582 | Western Lowland Gorilla | *Gorilla gorilla gorilla* | Dzangha Sangha, Central African Republic |
| 3.2009 | 6562 | Western Lowland Gorilla | *Gorilla gorilla gorilla* | Dzangha Sangha, Central African Republic |
| 4.2009 | 9887 | Western Lowland Gorilla | *Gorilla gorilla gorilla* | Dzangha Sangha, Central African Republic |
| 6.2009 | 4406 | Western Lowland Gorilla | *Gorilla gorilla gorilla* | Dzangha Sangha, Central African Republic |
| 7.2009 | 6353 | Western Lowland Gorilla | *Gorilla gorilla gorilla* | Dzangha Sangha, Central African Republic |
| 8.2009 | 7353 | Western Lowland Gorilla | *Gorilla gorilla gorilla* | Dzangha Sangha, Central African Republic |
| 9.1 | 8339 | Western Lowland Gorilla | *Gorilla gorilla gorilla* | Dzangha Sangha, Central African Republic |
| 9.114 | 19818 | Western Lowland Gorilla | *Gorilla gorilla gorilla* | Dzangha Sangha, Central African Republic |
| 9.2009 | 4666 | Western Lowland Gorilla | *Gorilla gorilla gorilla* | Dzangha Sangha, Central African Republic |
| 9.209 | 23064 | Western Lowland Gorilla | *Gorilla gorilla gorilla* | Dzangha Sangha, Central African Republic |
| 9.245 | 20642 | Western Lowland Gorilla | *Gorilla gorilla gorilla* | Dzangha Sangha, Central African Republic |
| 9.265 | 21501 | Western Lowland Gorilla | *Gorilla gorilla gorilla* | Dzangha Sangha, Central African Republic |
| 9.5 | 3072 | Western Lowland Gorilla | *Gorilla gorilla gorilla* | Dzangha Sangha, Central African Republic |
| 10.2009 | 10230 | Western Lowland Gorilla | *Gorilla gorilla gorilla* | Dzangha Sangha, Central African Republic |
| 11.1 | 3859 | Western Lowland Gorilla | *Gorilla gorilla gorilla* | Dzangha Sangha, Central African Republic |
| 11.103 | 2686 | Western Lowland Gorilla | *Gorilla gorilla gorilla* | Dzangha Sangha, Central African Republic |
| 11.105 | 7557 | Western Lowland Gorilla | *Gorilla gorilla gorilla* | Dzangha Sangha, Central African Republic |
| 11.106 | 2383 | Western Lowland Gorilla | *Gorilla gorilla gorilla* | Dzangha Sangha, Central African Republic |
| 11.108 | 6593 | Western Lowland Gorilla | *Gorilla gorilla gorilla* | Dzangha Sangha, Central African Republic |
| 11.109 | 156869 | Western Lowland Gorilla | *Gorilla gorilla gorilla* | Dzangha Sangha, Central African Republic |
| 11.11 | 3243 | Western Lowland Gorilla | *Gorilla gorilla gorilla* | Dzangha Sangha, Central African Republic |
| 11.117 | 2595 | Western Lowland Gorilla | *Gorilla gorilla gorilla* | Dzangha Sangha, Central African Republic |
| 11.118 | 3007 | Western Lowland Gorilla | *Gorilla gorilla gorilla* | Dzangha Sangha, Central African Republic |
| 11.119 | 3511 | Western Lowland Gorilla | *Gorilla gorilla gorilla* | Dzangha Sangha, Central African Republic |
| 11.12 | 3102 | Western Lowland Gorilla | *Gorilla gorilla gorilla* | Dzangha Sangha, Central African Republic |
| 11.123 | 4364 | Western Lowland Gorilla | *Gorilla gorilla gorilla* | Dzangha Sangha, Central African Republic |
| 11.124 | 3236 | Western Lowland Gorilla | *Gorilla gorilla gorilla* | Dzangha Sangha, Central African Republic |
| 11.13 | 26706 | Western Lowland Gorilla | *Gorilla gorilla gorilla* | Dzangha Sangha, Central African Republic |
| 11.133 | 6538 | Western Lowland Gorilla | *Gorilla gorilla gorilla* | Dzangha Sangha, Central African Republic |
| 11.134 | 7908 | Western Lowland Gorilla | *Gorilla gorilla gorilla* | Dzangha Sangha, Central African Republic |
| 11.136 | 10734 | Western Lowland Gorilla | *Gorilla gorilla gorilla* | Dzangha Sangha, Central African Republic |
| 11.139 | 5736 | Western Lowland Gorilla | *Gorilla gorilla gorilla* | Dzangha Sangha, Central African Republic |
| 11.14 | 11768 | Western Lowland Gorilla | *Gorilla gorilla gorilla* | Dzangha Sangha, Central African Republic |
| 11.141 | 9393 | Western Lowland Gorilla | *Gorilla gorilla gorilla* | Dzangha Sangha, Central African Republic |
| 11.142 | 5050 | Western Lowland Gorilla | *Gorilla gorilla gorilla* | Dzangha Sangha, Central African Republic |
| 11.143 | 5193 | Western Lowland Gorilla | *Gorilla gorilla gorilla* | Dzangha Sangha, Central African Republic |
| 11.145 | 3376 | Western Lowland Gorilla | *Gorilla gorilla gorilla* | Dzangha Sangha, Central African Republic |
| 11.147 | 7822 | Western Lowland Gorilla | *Gorilla gorilla gorilla* | Dzangha Sangha, Central African Republic |
| 11.148 | 3234 | Western Lowland Gorilla | *Gorilla gorilla gorilla* | Dzangha Sangha, Central African Republic |
| 11.149 | 3203 | Western Lowland Gorilla | *Gorilla gorilla gorilla* | Dzangha Sangha, Central African Republic |
| 11.15 | 33447 | Western Lowland Gorilla | *Gorilla gorilla gorilla* | Dzangha Sangha, Central African Republic |
| 11.152 | 4094 | Western Lowland Gorilla | *Gorilla gorilla gorilla* | Dzangha Sangha, Central African Republic |
| 11.169 | 7786 | Western Lowland Gorilla | *Gorilla gorilla gorilla* | Dzangha Sangha, Central African Republic |
| 11.17 | 8881 | Western Lowland Gorilla | *Gorilla gorilla gorilla* | Dzangha Sangha, Central African Republic |
| 11.173 | 8519 | Western Lowland Gorilla | *Gorilla gorilla gorilla* | Dzangha Sangha, Central African Republic |
| 11.18 | 30786 | Western Lowland Gorilla | *Gorilla gorilla gorilla* | Dzangha Sangha, Central African Republic |
| 11.19 | 13259 | Western Lowland Gorilla | *Gorilla gorilla gorilla* | Dzangha Sangha, Central African Republic |
| 11.193 | 8434 | Western Lowland Gorilla | *Gorilla gorilla gorilla* | Dzangha Sangha, Central African Republic |
| 11.194 | 7302 | Western Lowland Gorilla | *Gorilla gorilla gorilla* | Dzangha Sangha, Central African Republic |
| 11.196 | 5767 | Western Lowland Gorilla | *Gorilla gorilla gorilla* | Dzangha Sangha, Central African Republic |
| 11.197 | 8387 | Western Lowland Gorilla | *Gorilla gorilla gorilla* | Dzangha Sangha, Central African Republic |
| 11.2009 | 5289 | Western Lowland Gorilla | *Gorilla gorilla gorilla* | Dzangha Sangha, Central African Republic |
| 11.204 | 9508 | Western Lowland Gorilla | *Gorilla gorilla gorilla* | Dzangha Sangha, Central African Republic |
| 11.205 | 13265 | Western Lowland Gorilla | *Gorilla gorilla gorilla* | Dzangha Sangha, Central African Republic |
| 11.207 | 12478 | Western Lowland Gorilla | *Gorilla gorilla gorilla* | Dzangha Sangha, Central African Republic |
| 11.21 | 8646 | Western Lowland Gorilla | *Gorilla gorilla gorilla* | Dzangha Sangha, Central African Republic |
| 11.211 | 10408 | Western Lowland Gorilla | *Gorilla gorilla gorilla* | Dzangha Sangha, Central African Republic |
| 11.212 | 10848 | Western Lowland Gorilla | *Gorilla gorilla gorilla* | Dzangha Sangha, Central African Republic |
| 11.217 | 6785 | Western Lowland Gorilla | *Gorilla gorilla gorilla* | Dzangha Sangha, Central African Republic |
| 11.218 | 8865 | Western Lowland Gorilla | *Gorilla gorilla gorilla* | Dzangha Sangha, Central African Republic |
| 11.233 | 11037 | Western Lowland Gorilla | *Gorilla gorilla gorilla* | Dzangha Sangha, Central African Republic |
| 11.234 | 3293 | Western Lowland Gorilla | *Gorilla gorilla gorilla* | Dzangha Sangha, Central African Republic |
| 11.235 | 5351 | Western Lowland Gorilla | *Gorilla gorilla gorilla* | Dzangha Sangha, Central African Republic |
| 11.236 | 6425 | Western Lowland Gorilla | *Gorilla gorilla gorilla* | Dzangha Sangha, Central African Republic |
| 11.237 | 5734 | Western Lowland Gorilla | *Gorilla gorilla gorilla* | Dzangha Sangha, Central African Republic |
| 11.238 | 3720 | Western Lowland Gorilla | *Gorilla gorilla gorilla* | Dzangha Sangha, Central African Republic |
| 11.239 | 9149 | Western Lowland Gorilla | *Gorilla gorilla gorilla* | Dzangha Sangha, Central African Republic |
| 11.24 | 3904 | Western Lowland Gorilla | *Gorilla gorilla gorilla* | Dzangha Sangha, Central African Republic |
| 11.241 | 5622 | Western Lowland Gorilla | *Gorilla gorilla gorilla* | Dzangha Sangha, Central African Republic |
| 11.242 | 4964 | Western Lowland Gorilla | *Gorilla gorilla gorilla* | Dzangha Sangha, Central African Republic |
| 11.43 | 45322 | Western Lowland Gorilla | *Gorilla gorilla gorilla* | Dzangha Sangha, Central African Republic |
| 11.44 | 8227 | Western Lowland Gorilla | *Gorilla gorilla gorilla* | Dzangha Sangha, Central African Republic |
| 11.5 | 6468 | Western Lowland Gorilla | *Gorilla gorilla gorilla* | Dzangha Sangha, Central African Republic |
| 11.55 | 8401 | Western Lowland Gorilla | *Gorilla gorilla gorilla* | Dzangha Sangha, Central African Republic |
| 11.56 | 6822 | Western Lowland Gorilla | *Gorilla gorilla gorilla* | Dzangha Sangha, Central African Republic |
| 11.59 | 9716 | Western Lowland Gorilla | *Gorilla gorilla gorilla* | Dzangha Sangha, Central African Republic |
| 11.6 | 40420 | Western Lowland Gorilla | *Gorilla gorilla gorilla* | Dzangha Sangha, Central African Republic |
| 11.62 | 7043 | Western Lowland Gorilla | *Gorilla gorilla gorilla* | Dzangha Sangha, Central African Republic |
| 11.65 | 7522 | Western Lowland Gorilla | *Gorilla gorilla gorilla* | Dzangha Sangha, Central African Republic |
| 11.66 | 4576 | Western Lowland Gorilla | *Gorilla gorilla gorilla* | Dzangha Sangha, Central African Republic |
| 11.79 | 8255 | Western Lowland Gorilla | *Gorilla gorilla gorilla* | Dzangha Sangha, Central African Republic |
| 11.8 | 9215 | Western Lowland Gorilla | *Gorilla gorilla gorilla* | Dzangha Sangha, Central African Republic |
| 11.82 | 6534 | Western Lowland Gorilla | *Gorilla gorilla gorilla* | Dzangha Sangha, Central African Republic |
| 11.83 | 6967 | Western Lowland Gorilla | *Gorilla gorilla gorilla* | Dzangha Sangha, Central African Republic |
| 11.85 | 2771 | Western Lowland Gorilla | *Gorilla gorilla gorilla* | Dzangha Sangha, Central African Republic |
| 11.88 | 8464 | Western Lowland Gorilla | *Gorilla gorilla gorilla* | Dzangha Sangha, Central African Republic |
| 11.9 | 11269 | Western Lowland Gorilla | *Gorilla gorilla gorilla* | Dzangha Sangha, Central African Republic |
| 11.91 | 10133 | Western Lowland Gorilla | *Gorilla gorilla gorilla* | Dzangha Sangha, Central African Republic |
| 11.92 | 9920 | Western Lowland Gorilla | *Gorilla gorilla gorilla* | Dzangha Sangha, Central African Republic |
| 11.95 | 8956 | Western Lowland Gorilla | *Gorilla gorilla gorilla* | Dzangha Sangha, Central African Republic |
| 11.96 | 9764 | Western Lowland Gorilla | *Gorilla gorilla gorilla* | Dzangha Sangha, Central African Republic |
| 12.102 | 5464 | Western Lowland Gorilla | *Gorilla gorilla gorilla* | Dzangha Sangha, Central African Republic |
| 12.103 | 4919 | Western Lowland Gorilla | *Gorilla gorilla gorilla* | Dzangha Sangha, Central African Republic |
| 12.104 | 5121 | Western Lowland Gorilla | *Gorilla gorilla gorilla* | Dzangha Sangha, Central African Republic |
| 12.106 | 5564 | Western Lowland Gorilla | *Gorilla gorilla gorilla* | Dzangha Sangha, Central African Republic |
| 12.107 | 8146 | Western Lowland Gorilla | *Gorilla gorilla gorilla* | Dzangha Sangha, Central African Republic |
| 12.108 | 4765 | Western Lowland Gorilla | *Gorilla gorilla gorilla* | Dzangha Sangha, Central African Republic |
| 12.109 | 4473 | Western Lowland Gorilla | *Gorilla gorilla gorilla* | Dzangha Sangha, Central African Republic |
| 12.11 | 5765 | Western Lowland Gorilla | *Gorilla gorilla gorilla* | Dzangha Sangha, Central African Republic |
| 12.111 | 4279 | Western Lowland Gorilla | *Gorilla gorilla gorilla* | Dzangha Sangha, Central African Republic |
| 12.112 | 6230 | Western Lowland Gorilla | *Gorilla gorilla gorilla* | Dzangha Sangha, Central African Republic |
| 12.113 | 5493 | Western Lowland Gorilla | *Gorilla gorilla gorilla* | Dzangha Sangha, Central African Republic |
| 12.114 | 8777 | Western Lowland Gorilla | *Gorilla gorilla gorilla* | Dzangha Sangha, Central African Republic |
| 12.115 | 5183 | Western Lowland Gorilla | *Gorilla gorilla gorilla* | Dzangha Sangha, Central African Republic |
| 12.116 | 3983 | Western Lowland Gorilla | *Gorilla gorilla gorilla* | Dzangha Sangha, Central African Republic |
| 12.153 | 4565 | Western Lowland Gorilla | *Gorilla gorilla gorilla* | Dzangha Sangha, Central African Republic |
| 12.155 | 5559 | Western Lowland Gorilla | *Gorilla gorilla gorilla* | Dzangha Sangha, Central African Republic |
| 12.199 | 4055 | Western Lowland Gorilla | *Gorilla gorilla gorilla* | Dzangha Sangha, Central African Republic |
| 12.2 | 4372 | Western Lowland Gorilla | *Gorilla gorilla gorilla* | Dzangha Sangha, Central African Republic |
| 12.2009 | 5686 | Western Lowland Gorilla | *Gorilla gorilla gorilla* | Dzangha Sangha, Central African Republic |
| 12.217 | 3238 | Western Lowland Gorilla | *Gorilla gorilla gorilla* | Dzangha Sangha, Central African Republic |
| 12.218 | 3810 | Western Lowland Gorilla | *Gorilla gorilla gorilla* | Dzangha Sangha, Central African Republic |
| 12.227 | 4832 | Western Lowland Gorilla | *Gorilla gorilla gorilla* | Dzangha Sangha, Central African Republic |
| 12.228 | 4906 | Western Lowland Gorilla | *Gorilla gorilla gorilla* | Dzangha Sangha, Central African Republic |
| 12.229 | 5901 | Western Lowland Gorilla | *Gorilla gorilla gorilla* | Dzangha Sangha, Central African Republic |
| 12.23 | 7532 | Western Lowland Gorilla | *Gorilla gorilla gorilla* | Dzangha Sangha, Central African Republic |
| 12.231 | 7874 | Western Lowland Gorilla | *Gorilla gorilla gorilla* | Dzangha Sangha, Central African Republic |
| 12.232 | 6118 | Western Lowland Gorilla | *Gorilla gorilla gorilla* | Dzangha Sangha, Central African Republic |
| 12.233 | 5951 | Western Lowland Gorilla | *Gorilla gorilla gorilla* | Dzangha Sangha, Central African Republic |
| 12.234 | 15509 | Western Lowland Gorilla | *Gorilla gorilla gorilla* | Dzangha Sangha, Central African Republic |
| 12.236 | 8628 | Western Lowland Gorilla | *Gorilla gorilla gorilla* | Dzangha Sangha, Central African Republic |
| 12.237 | 18535 | Western Lowland Gorilla | *Gorilla gorilla gorilla* | Dzangha Sangha, Central African Republic |
| 12.28 | 37553 | Western Lowland Gorilla | *Gorilla gorilla gorilla* | Dzangha Sangha, Central African Republic |
| 12.29 | 3461 | Western Lowland Gorilla | *Gorilla gorilla gorilla* | Dzangha Sangha, Central African Republic |
| 12.3 | 2743 | Western Lowland Gorilla | *Gorilla gorilla gorilla* | Dzangha Sangha, Central African Republic |
| 12.3 | 3518 | Western Lowland Gorilla | *Gorilla gorilla gorilla* | Dzangha Sangha, Central African Republic |
| 12.32 | 3810 | Western Lowland Gorilla | *Gorilla gorilla gorilla* | Dzangha Sangha, Central African Republic |
| 12.36 | 3650 | Western Lowland Gorilla | *Gorilla gorilla gorilla* | Dzangha Sangha, Central African Republic |
| 12.4 | 6647 | Western Lowland Gorilla | *Gorilla gorilla gorilla* | Dzangha Sangha, Central African Republic |
| 12.42 | 2420 | Western Lowland Gorilla | *Gorilla gorilla gorilla* | Dzangha Sangha, Central African Republic |
| 12.43 | 3114 | Western Lowland Gorilla | *Gorilla gorilla gorilla* | Dzangha Sangha, Central African Republic |
| 12.47 | 27505 | Western Lowland Gorilla | *Gorilla gorilla gorilla* | Dzangha Sangha, Central African Republic |
| 12.48 | 4474 | Western Lowland Gorilla | *Gorilla gorilla gorilla* | Dzangha Sangha, Central African Republic |
| 12.49 | 4672 | Western Lowland Gorilla | *Gorilla gorilla gorilla* | Dzangha Sangha, Central African Republic |
| 12.5 | 4586 | Western Lowland Gorilla | *Gorilla gorilla gorilla* | Dzangha Sangha, Central African Republic |
| 12.51 | 3493 | Western Lowland Gorilla | *Gorilla gorilla gorilla* | Dzangha Sangha, Central African Republic |
| 12.52 | 3083 | Western Lowland Gorilla | *Gorilla gorilla gorilla* | Dzangha Sangha, Central African Republic |
| 12.53 | 5326 | Western Lowland Gorilla | *Gorilla gorilla gorilla* | Dzangha Sangha, Central African Republic |
| 12.54 | 8016 | Western Lowland Gorilla | *Gorilla gorilla gorilla* | Dzangha Sangha, Central African Republic |
| 12.55 | 6034 | Western Lowland Gorilla | *Gorilla gorilla gorilla* | Dzangha Sangha, Central African Republic |
| 12.98 | 9249 | Western Lowland Gorilla | *Gorilla gorilla gorilla* | Dzangha Sangha, Central African Republic |
| 13.2009 | 12951 | Western Lowland Gorilla | *Gorilla gorilla gorilla* | Dzangha Sangha, Central African Republic |
| 14.2009 | 6791 | Western Lowland Gorilla | *Gorilla gorilla gorilla* | Dzangha Sangha, Central African Republic |
| 15.2009 | 11231 | Western Lowland Gorilla | *Gorilla gorilla gorilla* | Dzangha Sangha, Central African Republic |
| 16.2009 | 5412 | Western Lowland Gorilla | *Gorilla gorilla gorilla* | Dzangha Sangha, Central African Republic |
| 17.2009 | 6224 | Western Lowland Gorilla | *Gorilla gorilla gorilla* | Dzangha Sangha, Central African Republic |
| 18.2009 | 7479 | Western Lowland Gorilla | *Gorilla gorilla gorilla* | Dzangha Sangha, Central African Republic |
| 20.2009 | 9096 | Western Lowland Gorilla | *Gorilla gorilla gorilla* | Dzangha Sangha, Central African Republic |
| 20.2011 | 12213 | Western Lowland Gorilla | *Gorilla gorilla gorilla* | Dzangha Sangha, Central African Republic |
| 21.2009 | 5966 | Western Lowland Gorilla | *Gorilla gorilla gorilla* | Dzangha Sangha, Central African Republic |
| 21.2011 | 12205 | Western Lowland Gorilla | *Gorilla gorilla gorilla* | Dzangha Sangha, Central African Republic |
| 22.2009 | 5270 | Western Lowland Gorilla | *Gorilla gorilla gorilla* | Dzangha Sangha, Central African Republic |
| 22.2011 | 6490 | Western Lowland Gorilla | *Gorilla gorilla gorilla* | Dzangha Sangha, Central African Republic |
| 23.2009 | 5316 | Western Lowland Gorilla | *Gorilla gorilla gorilla* | Dzangha Sangha, Central African Republic |
| 23.2011 | 11042 | Western Lowland Gorilla | *Gorilla gorilla gorilla* | Dzangha Sangha, Central African Republic |
| 24.2009 | 6051 | Western Lowland Gorilla | *Gorilla gorilla gorilla* | Dzangha Sangha, Central African Republic |
| 25.2009 | 5519 | Western Lowland Gorilla | *Gorilla gorilla gorilla* | Dzangha Sangha, Central African Republic |
| 26.2009 | 5521 | Western Lowland Gorilla | *Gorilla gorilla gorilla* | Dzangha Sangha, Central African Republic |
| 27.2009 | 4919 | Western Lowland Gorilla | *Gorilla gorilla gorilla* | Dzangha Sangha, Central African Republic |
| 28.2009 | 5224 | Western Lowland Gorilla | *Gorilla gorilla gorilla* | Dzangha Sangha, Central African Republic |
| 31.2011 | 3473 | Western Lowland Gorilla | *Gorilla gorilla gorilla* | Dzangha Sangha, Central African Republic |
| 31.2012 | 2703 | Western Lowland Gorilla | *Gorilla gorilla gorilla* | Dzangha Sangha, Central African Republic |
| 32.2009 | 4908 | Western Lowland Gorilla | *Gorilla gorilla gorilla* | Dzangha Sangha, Central African Republic |
| 34.2009 | 4761 | Western Lowland Gorilla | *Gorilla gorilla gorilla* | Dzangha Sangha, Central African Republic |
| 35.2009 | 6371 | Western Lowland Gorilla | *Gorilla gorilla gorilla* | Dzangha Sangha, Central African Republic |
| 36.2009 | 5498 | Western Lowland Gorilla | *Gorilla gorilla gorilla* | Dzangha Sangha, Central African Republic |
| 37.2009 | 5085 | Western Lowland Gorilla | *Gorilla gorilla gorilla* | Dzangha Sangha, Central African Republic |
| 38.2009 | 7243 | Western Lowland Gorilla | *Gorilla gorilla gorilla* | Dzangha Sangha, Central African Republic |
| 39.2009 | 13113 | Western Lowland Gorilla | *Gorilla gorilla gorilla* | Dzangha Sangha, Central African Republic |
| 57.2011 | 13058 | Western Lowland Gorilla | *Gorilla gorilla gorilla* | Dzangha Sangha, Central African Republic |
| 146.2011 | 5475 | Western Lowland Gorilla | *Gorilla gorilla gorilla* | Dzangha Sangha, Central African Republic |
| 146.2012 | 3950 | Western Lowland Gorilla | *Gorilla gorilla gorilla* | Dzangha Sangha, Central African Republic |
| 154.2011 | 3116 | Western Lowland Gorilla | *Gorilla gorilla gorilla* | Dzangha Sangha, Central African Republic |
| 154.2012 | 1844 | Western Lowland Gorilla | *Gorilla gorilla gorilla* | Dzangha Sangha, Central African Republic |
| 192.2011 | 9281 | Western Lowland Gorilla | *Gorilla gorilla gorilla* | Dzangha Sangha, Central African Republic |
| 09.4i | 16633 | Western Lowland Gorilla | *Gorilla gorilla gorilla* | Dzangha Sangha, Central African Republic |
| 12.57A | 10521 | Western Lowland Gorilla | *Gorilla gorilla gorilla* | Dzangha Sangha, Central African Republic |
| 12.58A | 11369 | Western Lowland Gorilla | *Gorilla gorilla gorilla* | Dzangha Sangha, Central African Republic |
| 12.60A | 7515 | Western Lowland Gorilla | *Gorilla gorilla gorilla* | Dzangha Sangha, Central African Republic |
| 12.65A | 28580 | Western Lowland Gorilla | *Gorilla gorilla gorilla* | Dzangha Sangha, Central African Republic |
| 12.68A | 12628 | Western Lowland Gorilla | *Gorilla gorilla gorilla* | Dzangha Sangha, Central African Republic |
| 12.70A | 7136 | Western Lowland Gorilla | *Gorilla gorilla gorilla* | Dzangha Sangha, Central African Republic |
| 12.72A | 10720 | Western Lowland Gorilla | *Gorilla gorilla gorilla* | Dzangha Sangha, Central African Republic |
| E.107.2009 | 6387 | Western Lowland Gorilla | *Gorilla gorilla gorilla* | Dzangha Sangha, Central African Republic |
| E.148.2009 | 7361 | Western Lowland Gorilla | *Gorilla gorilla gorilla* | Dzangha Sangha, Central African Republic |
| E.173.2009 | 4949 | Western Lowland Gorilla | *Gorilla gorilla gorilla* | Dzangha Sangha, Central African Republic |
| E.174.2009 | 4195 | Western Lowland Gorilla | *Gorilla gorilla gorilla* | Dzangha Sangha, Central African Republic |
| E.193.2009 | 6378 | Western Lowland Gorilla | *Gorilla gorilla gorilla* | Dzangha Sangha, Central African Republic |
| E.245.2009 | 3658 | Western Lowland Gorilla | *Gorilla gorilla gorilla* | Dzangha Sangha, Central African Republic |
| E.265.2009 | 5405 | Western Lowland Gorilla | *Gorilla gorilla gorilla* | Dzangha Sangha, Central African Republic |
| E.283.2009 | 12913 | Western Lowland Gorilla | *Gorilla gorilla gorilla* | Dzangha Sangha, Central African Republic |
| Hu.125 | 6581 | Western Researcher at CAR | *Homo sapiens* | Czech at Dzanga Sangha, , Central African Republic |
| Hu.126 | 10182 | Western Researcher at CAR | *Homo sapiens* | Czech at Dzanga Sangha, Central African Republic |
| Hu.127 | 18744 | Western Researcher at CAR | *Homo sapiens* | British at Dzanga Sangha, Central African Republic |
| Hu.509 | 18045 | Western Researcher at CAR | *Homo sapiens* | US American at Dzanga Sangha, Central African Republic |
| Hu.510 | 6424 | Western Researcher at CAR | *Homo sapiens* | US American at Dzanga Sangha, Central African Republic |
