# Supplementary figures and images for: Plasticity in the Human Gut Microbiome Defies Evolutionary Constraints
Source: mSphere. 2019 Jul 31;4(4):e00271-19. doi: 10.1128/mSphere.00271-19 (PMC6669335; doi:10.1128/mSphere.00271-19)

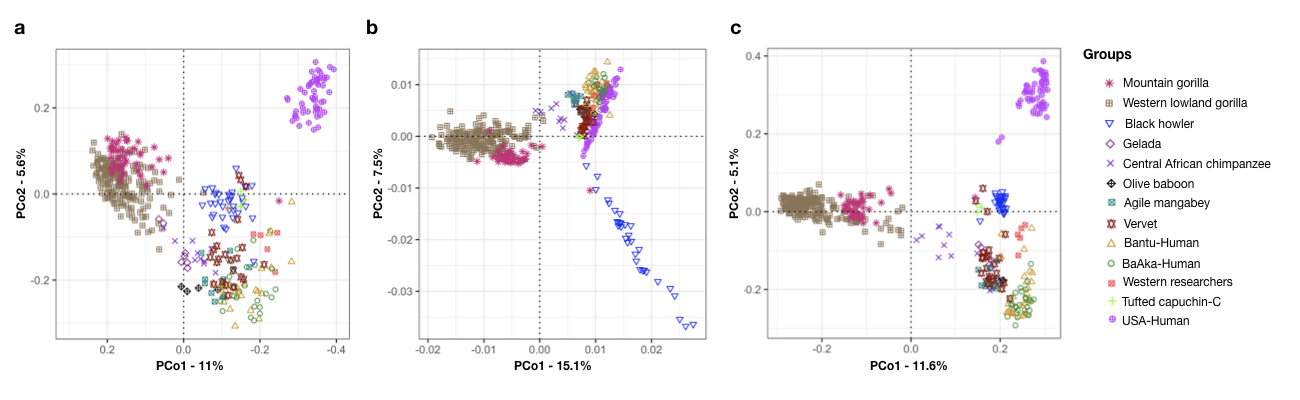

Supplement: FIG S1 [file mSphere.00271-19-sf001.tiff]

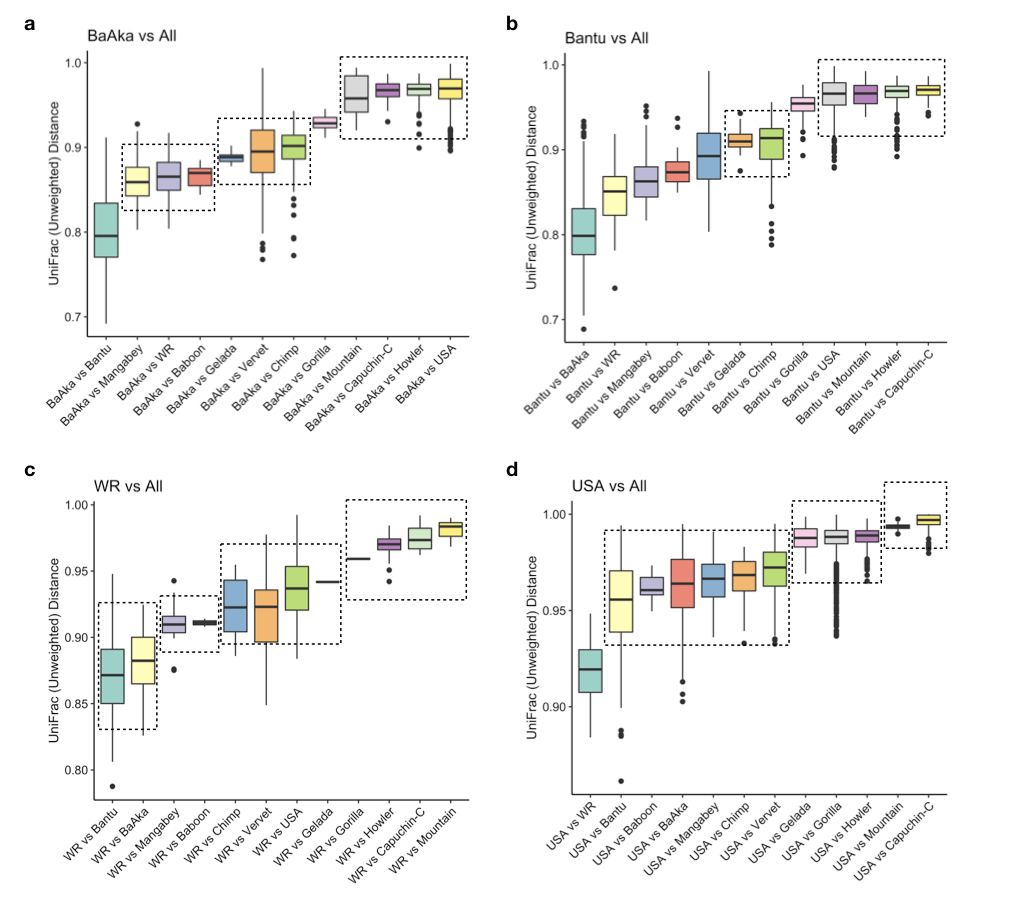

Supplement: FIG S2 [file mSphere.00271-19-sf002.tif]

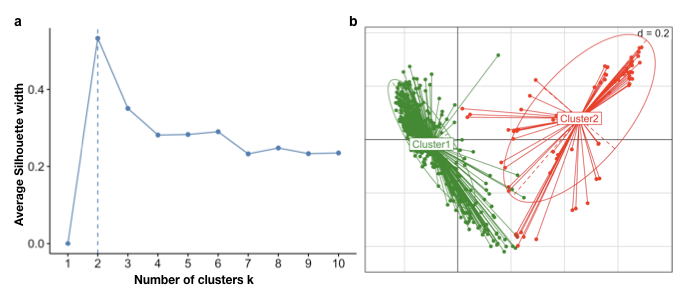

Supplement: FIG S3 [file mSphere.00271-19-sf003.tif]

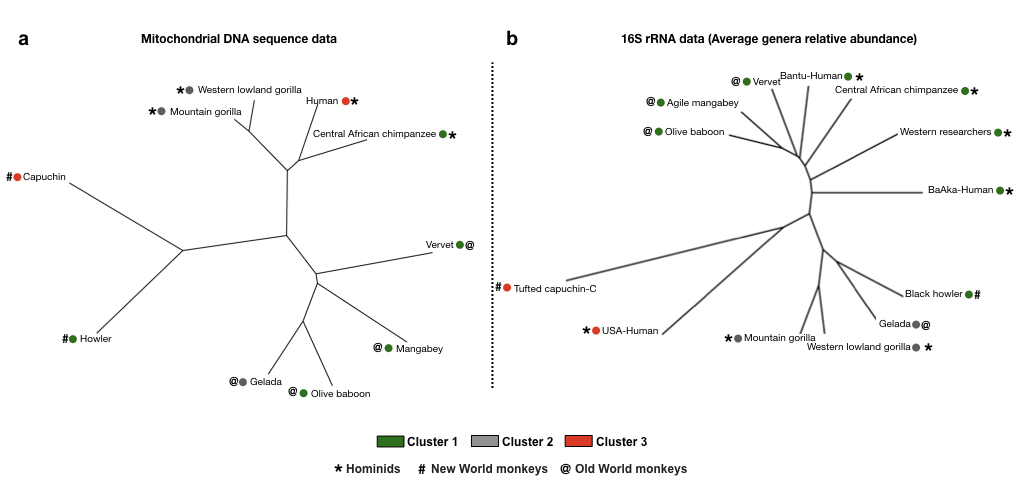

Supplement: FIG S4 [file mSphere.00271-19-sf004.tiff]

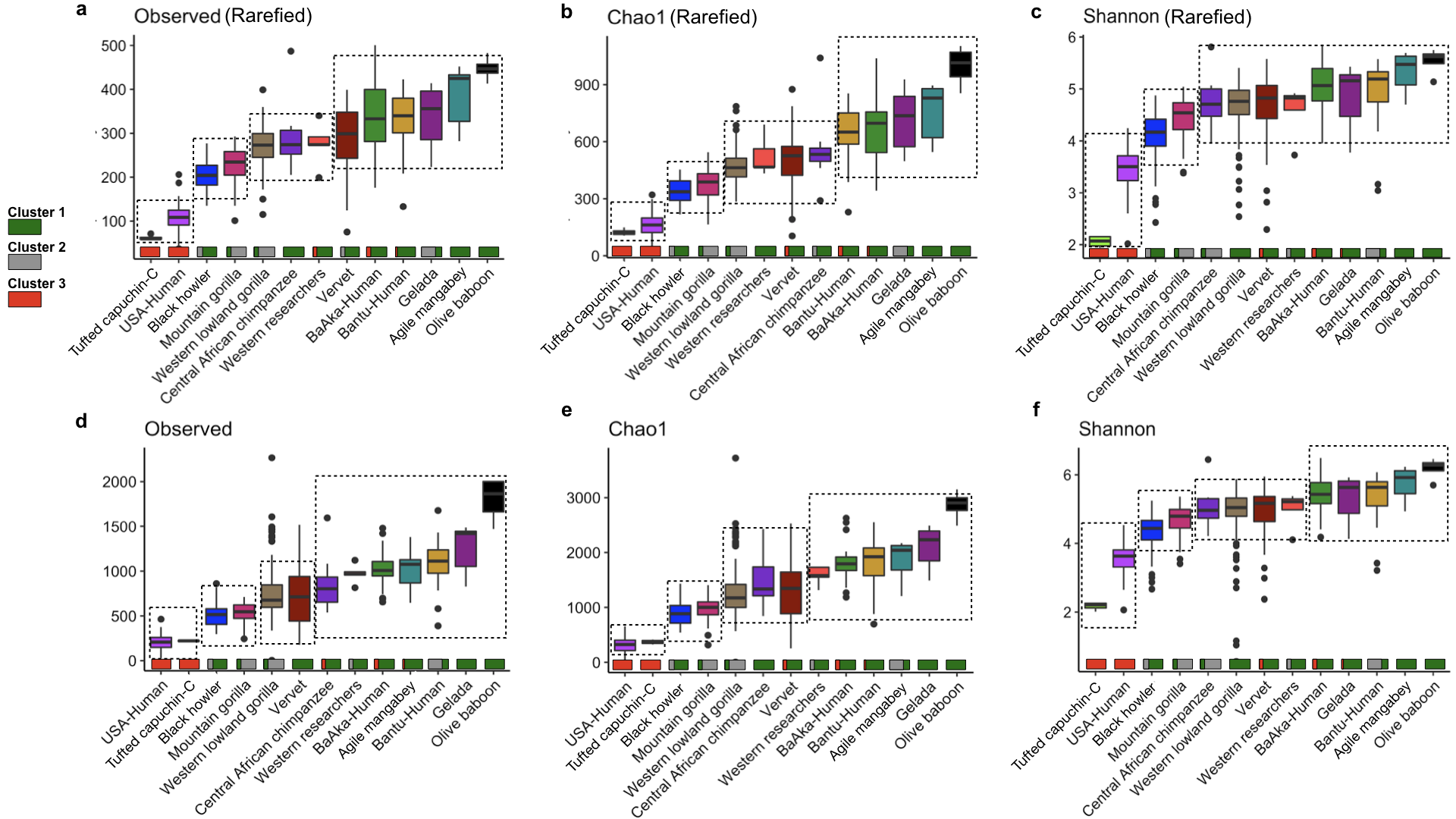

Supplement: FIG S5 [file mSphere.00271-19-sf005.tiff]

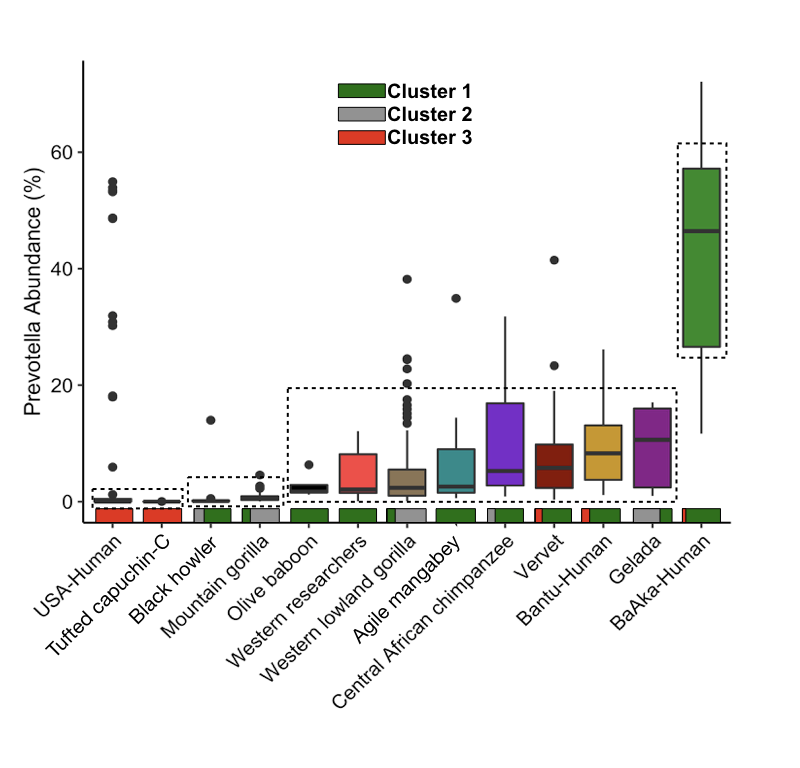

Supplement: FIG S6 [file mSphere.00271-19-sf006.tiff]
