# Supplementary material for: Plasticity in the Human Gut Microbiome Defies Evolutionary Constraints
Source: mSphere. 2019 Jul 31;4(4):e00271-19. doi: 10.1128/mSphere.00271-19 (PMC6669335; doi:10.1128/mSphere.00271-19)
Supplement: TABLE S2 [file mSphere.00271-19-st002.docx]

| **Taxa** | **Mean Relative Abundance (%)** | | | **MeanDecreaseAccuracy** | **Indval Score** |
| --- | --- | --- | --- | --- | --- |
|  | **Clust.1** | **Clust.2** | **Clus.3** |  |  |
| k__Bacteria.p__Bacteroidetes.c__Bacteroidia.o__Bacteroidales.f__Bacteroidaceae.g__Bacteroides | 0.703034 | 0.09888 | 44.9999 | 13.23079534 | 0.97 |
| k__Bacteria.p__Spirochaetes.c__Spirochaetes.o__Sphaerochaetales.f__Sphaerochaetaceae.g__Sphaerochaeta | 0.204906 | 2.13856 | 2.09E-03 | 15.37043396 | 0.89 |
| k__Bacteria.p__Firmicutes.c__Erysipelotrichi.o__Erysipelotrichales.f__Erysipelotrichaceae.g__RFN20 | 0.147398 | 0.93355 | 2.40E-04 | 15.8671971 | 0.85 |
| k__Bacteria.p__Chloroflexi.c__Anaerolineae.o__Anaerolineales.f__Anaerolinaceae.g__SHD.231 | 0.835976 | 4.43141 | 0.01918 | 16.07889593 | 0.83 |
| k__Bacteria.p__Bacteroidetes.c__Bacteroidia.o__Bacteroidales.f__Porphyromonadaceae.g__Parabacteroides | 0.25064 | 0.0484 | 2.56383 | 9.476088587 | 0.8 |
| k__Bacteria.p__Actinobacteria.c__Coriobacteriia.o__Coriobacteriales.f__Coriobacteriaceae.g__Adlercreutzia | 0.024718 | 0.3043 | 0 | 11.88529454 | 0.78 |
| k__Bacteria.p__Firmicutes.c__Clostridia.o__Clostridiales.f__.Mogibacteriaceae..g__Mogibacterium | 0.070983 | 0.412 | 0.00088 | 10.81374802 | 0.77 |
| k__Bacteria.p__Spirochaetes.c__Spirochaetes.o__Spirochaetales.f__Spirochaetaceae.g__Treponema | 0.771964 | 2.48734 | 0.00519 | 12.30893614 | 0.75 |
| k__Bacteria.p__TM7.c__TM7.3.o__CW040.f__F16.g__ | 0.057991 | 0.62139 | 0.00026 | 11.26877666 | 0.75 |
| k__Bacteria.p__Actinobacteria.c__Coriobacteriia.o__Coriobacteriales.f__Coriobacteriaceae.g__ | 1.568739 | 4.35521 | 0.00139 | 16.37006138 | 0.73 |
| k__Bacteria.p__Fibrobacteres.c__Fibrobacteria.o__Fibrobacterales.f__Fibrobacteraceae.g__Fibrobacter | 0.038354 | 0.29263 | 0.0018 | 7.167004022 | 0.71 |
| k__Bacteria.p__Proteobacteria.c__Betaproteobacteria.o__Burkholderiales.f__Alcaligenaceae.g__Sutterella | 0.308418 | 0.32245 | 2.02798 | 9.025601424 | 0.66 |
| k__Bacteria.p__Firmicutes.c__Clostridia.o__Clostridiales.f__Lachnospiraceae.g__Butyrivibrio | 0.249491 | 0.53095 | 1.82E-04 | 10.57697977 | 0.64 |
| k__Bacteria.p__Firmicutes.c__Clostridia.o__Clostridiales.f__Ruminococcaceae.g__Faecalibacterium | 2.195243 | 0.21146 | 0.76003 | 13.119482 | 0.63 |
| k__Bacteria.p__Firmicutes.c__Erysipelotrichi.o__Erysipelotrichales.f__Erysipelotrichaceae.g__Bulleidia | 0.400023 | 0.83421 | 6.57E-03 | 10.34143355 | 0.62 |
| Unassigned.Other.Other.Other.Other.Other | 5.432683 | 13.4885 | 1.17305 | 17.1983703 | 0.61 |
| k__Bacteria.p__Firmicutes.c__Clostridia.o__Clostridiales.f__Lachnospiraceae.g__Coprococcus | 2.252958 | 0.84354 | 0.52758 | 9.61026354 | 0.61 |
| k__Bacteria.p__Firmicutes.c__Clostridia.o__Clostridiales.f__Lachnospiraceae.g__Lachnospira | 1.658902 | 0.09333 | 0.54682 | 7.877104382 | 0.61 |
| k__Bacteria.p__Firmicutes.c__Clostridia.o__Clostridiales.f__Clostridiaceae.g__Clostridium | 4.773172 | 3.10111 | 0.14147 | 12.81891854 | 0.58 |
| k__Bacteria.p__Proteobacteria.c__Alphaproteobacteria.o__RF32.f__.g__ | 0.390869 | 0.05438 | 1.62E-03 | 8.568068378 | 0.57 |
| k__Bacteria.p__Firmicutes.c__Bacilli.o__Lactobacillales.f__Streptococcaceae.g__Streptococcus | 0.173888 | 0.10942 | 1.22015 | 1.202966351 | 0.55 |
| k__Bacteria.p__Firmicutes.c__Erysipelotrichi.o__Erysipelotrichales.f__Erysipelotrichaceae.g__p.75.a5 | 0.280606 | 1.04457 | 0.5892 | 14.49854288 | 0.53 |
| k__Bacteria.p__Bacteroidetes.c__Bacteroidia.o__Bacteroidales.f__.Odoribacteraceae..g__Odoribacter | 2.99E-03 | 2.97E-04 | 0.23003 | 6.552629903 | 0.53 |
| k__Bacteria.p__Lentisphaerae.c__.Lentisphaeria..o__Victivallales.f__Victivallaceae.g__ | 0.23098 | 3.49E-03 | 3.92E-03 | 9.56179232 | 0.51 |
| k__Bacteria.p__Bacteroidetes.c__Bacteroidia.o__Bacteroidales.f__Prevotellaceae.g__Prevotella | 12.06407 | 3.56572 | 8.04878 | 9.568128848 | 0.5 |
